# A Multimodal MR Imaging Study of the Effect of Hippocampal Damage on Affective and Cognitive Functions in a Rat Model of Chronic Exposure to a Plateau Environment

**DOI:** 10.1007/s11064-021-03498-5

**Published:** 2022-01-04

**Authors:** Dongyong Zhu, Bo He, Mengdi Zhang, Yixuan Wan, Ruibin Liu, Lei Wang, Yi Zhang, Yunqing Li, Fabao Gao

**Affiliations:** 1 Department of Radiology, West China Hospital, Sichuan University, No. 37 Guoxue Road, Chengdu, 610041 China; 2 Department of Biomedical Engineering, College of Biomedical Engineering and Instrument Science, Zhejiang University, Hangzhou, 310030 China; 3 Molecular Imaging Center, West China Hospital, Sichuan University, Chengdu, 610041 China; 4 Department of Anatomy and KK Leung Brain Research Centre, The Fourth Military Medical University, Xi’an, 710032 China

**Keywords:** Voxel-based morphometry, Chemical exchange saturation transfer, Dynamic contrast-enhanced MR imaging, Animal model, Hippocampal damage, Plateau hypoxia

## Abstract

Prolonged exposure to high altitudes above 2500 m above sea level (a.s.l.) can cause cognitive and behavioral dysfunctions. Herein, we sought to investigate the effects of chronic exposure to plateau hypoxia on the hippocampus in a rat model by using voxel-based morphometry, creatine chemical exchange saturation transfer (CrCEST) and dynamic contrast-enhanced MR imaging techniques. 58 healthy 4-week-old male rats were randomized into plateau hypoxia rats (H group) as the experimental group and plain rats (P group) as the control group. H group rats were transported from Chengdu (500 m a.s.l.), a city in a plateau located in southwestern China, to the Qinghai–Tibet Plateau (4250 m a.s.l.), Yushu, China, and then fed for 8 months there, while P group rats were fed in Chengdu (500 m a.s.l.), China. After 8 months of exposure to plateau hypoxia, open-field and elevated plus maze tests revealed that the anxiety-like behavior of the H group rats was more serious than that of the P group rats, and the Morris water maze test revealed impaired spatial memory function in the H group rats. Multimodal MR imaging analysis revealed a decreased volume of the regional gray matter, lower CrCEST contrast and higher transport coefficient Ktrans in the hippocampus compared with the P group rats. Further correlation analysis found associations of quantitative MRI parameters of the hippocampus with the behavioral performance of H group rats. In this study, we validated the viability of using noninvasive multimodal MR imaging techniques to evaluate the effects of chronic exposure to a plateau hypoxic environment on the hippocampus.

**Supplementary Information:**

The online version contains supplementary material available at 10.1007/s11064-021-03498-5.

## Introduction

Hypobaric hypoxic exposure is a hazard that can lead to severe abnormalities in physiological and psychological functions due to strong stress responses of the body. Most of the previous studies established high-altitude hypoxic animal models using an animal decompression chamber. Simulated hypobaric hypoxia is often needed to bring the chamber down to sea level to replenish food and water at regular intervals, which is apparently different from genuine plateau environments. In this study, we established a rat brain damage model under chronic exposure to a natural plateau hypoxic environment. It has been widely reported that prolonged exposure to high-altitude hypobaric hypoxia may induce deficits in learning and memory [1–3]. As the hippocampus is closely associated with explicit memory, especially spatial learning and memory, any insult to this region can affect cognitive functions [4]. Studies of the differential susceptibility of brain regions to hypobaric hypoxia suggest that the hippocampus is more susceptible to hypoxic stress than the cerebellum, the cortex and the striatum [5, 6]. Hypobaric hypoxia-induced stress can cause stress in hippocampal oxidation, synaptic dysfunction, neurodegeneration and apoptosis and thus result in learning and memory deficits [7–10]. Therefore, it is necessary to research and develop effective and widely available biomarkers for the early detection of hippocampal damage.

Among the many techniques applicable to such early detection are multimodal MR imaging techniques [11], such as voxel-based morphometry (VBM), chemical exchange saturation transfer (CEST) imaging and dynamic contrast-enhanced MR imaging (DCE–MRI). They provide noninvasive means to detect morphological alterations in the gray matter, macromolecular protein level and cerebrovascular microcirculation. VBM, based on voxel differences in tissue classification, provides an effective neuroimaging approach to the investigation of subtle and discrete changes in regional gray matter volume (rGMV)/density in the entire brain [12]. A previous neuroimaging study [13] of high-altitude sojourners revealed significant gray matter loss and atrophy in several cerebral regions, including the cortex, hippocampus and striatum, suggesting that hypobaric hypoxia exposure may result in neuronal damage and impair cognitive functions [14]. However, to date, it has remained unknown whether VBM-detected changes in the regional gray matter of the hippocampus are directly linked to memory functions and mood.

In addition to VBM is CEST imaging, which has also been reported to be sensitive to macromolecular metabolites and proteins in tissues [15, 16]. Creatine CEST (CrCEST) contrast, enabling Cr imaging with high sensitivity and good spatial resolution [17], has been used as an in vivo tool for measuring free Cr because it can visualize and quantify oxidative phosphorylation capacity [18]. Hypobaric hypoxia exposure may obstruct the energy metabolic pathway and result in mitochondrial dysfunction and energy disorders [19], thus damaging and even killing cells in the brain [20]. In the present study, we sought to investigate the feasibility of the CrCEST for investigating changes in Cr in the brain and to evaluate the effect of hippocampal damage on affective and cognitive behaviors in a rat model of chronic exposure to hypobaric hypoxia.

DCE–MRI, the third multimodal MRI technique for the early detection of hippocampal damage, can quantify permeability in vivo even in small animals by assessing the diffusive transport of gadopentetate dimeglumine across the blood–brain barrier (BBB) [21, 22]. Previous studies have reported that chronic exposure to hypobaric hypoxia can biochemically alter the BBB [23] and increase its permeability [24]. To fill this niche in DCE–MRI, it is necessary to explore the influence of chronic exposure to hypobaric hypoxia on cerebral microvessels by using DCE–MRI to assess BBB permeability.

To the best of our knowledge, few studies have integrated the VBM, CrCEST and DCE–MRI techniques to evaluate the effects of plateau hypoxia exposure on the brain in a comprehensive manner. It is under such circumstances and to fill the abovementioned niches that this study is conducted to use the above three multimodal MR imaging techniques to detect alterations in rGMV, free Cr concentration, and BBB function of the brain in a rat model chronically exposed to a plateau hypoxic environment and thus to verify whether the regional changes in the brain could reveal the mechanisms underlying hypobaric hypoxia-induced cognitive and behavioral dysfunctions. Given that the hippocampus is more susceptible to hypoxic stress than other regions of the brain, this study was focused on changes in imaging parameters of this region.

## Materials and Methods

### Ethical Approval and Animals

58 healthy male Sprague–Dawley rats weighing 100 g (~ 4 weeks old) were purchased from Chengdu Dashuo Biological Technology Co., Ltd., Chengdu, China, and used in the present study. This study followed the protocols of and was approved by the Experimental Animal Ethics Committee of the West China Hospital, Sichuan University, Chengdu, China (Approval No. IACUC#20211239A). All experiments were executed in compliance with the relevant guidelines and regulations.

### Experimental Procedure

**Fig. 1:**
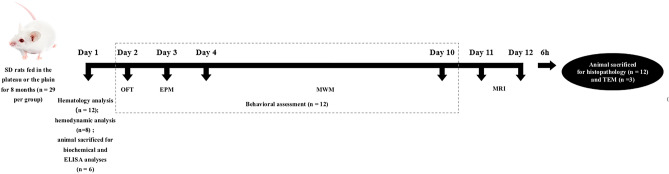
Experimental flowchart. The open field test (OFT) was conducted on the 2nd day, and the elevated plus-maze (EPM) test was conducted on the 3rd day to detect potential emotional changes (e.g., anxiety-like behaviors) in the H and P group rats. Performance of spatial learning and memory was measured with the Morris water maze (MWM) task on the 4th–10th days

The animals were randomized into two groups, including a plain group (P group rats) reared at an altitude of 500 m above sea level (a.s.l.) for 8 months and a plateau hypoxia group (H group rats) reared on the Qinghai-Tibet Plateau, Yushu, China, at an altitude of 4250 m a.s.l. for 8 months (n = 29 for each group). All the animals were fed standard laboratory feed and water readily available in all cages (6 rats in each cage) at an environmental temperature of 22–24 °C and with a humidity of 50–60%. The whole experimental flowchart is shown in Fig. 1.

### Analysis of Blood Cell Content

After 8 months, blood samples were collected from the tail vein under isoflurane anesthesia (n = 12/per group). For routine blood analysis, coccygeal vein blood (1 ml) was collected with a Mindray automatic hematology analyzer (BC-2800vet, Shenzhen, China).

### Hemodynamic Analysis

The rats (n = 8/per group) were subjected to measurement of mean pulmonary arterial pressure and mean systemic arterial pressure under anesthesia produced by an intraperitoneal injection of urethane (1.0 g/kg body weight) based on the rat weight as previously described [25]. The pressure transducer was connected to the Biopac MP150, a data acquisition system, using a polystyrene microcatheter thoroughly soaked in heparin brine. After anesthesia, the right external jugular vein was shunted carefully, and the catheter was slowly pushed to the lower part. The catheter was positioned in accordance with the waveform until the typical pulmonary pressure waveform appeared. After stabilizing for 5 min, the mean pulmonary pressure was recorded.

### Analysis of Oxidative Stress Parameters

The rats in both groups (n = 6/group) were deeply anesthetized with isoflurane and then decapitated. Following the manufacturer’s manual, we assessed the level of superoxide dismutase (SOD) (Cat. No. KTB1030), malondialdehyde (MDA) (KTB1050), glutathione peroxidase (GSH-Px) (KTB0640), glutathione (GSH) (KTB1600) and catalase (CAT) (KTB1040) in the hippocampus and the cortex with commercial assay kits purchased from Abbkine Scientific Co., Ltd., (CA, USA). The absorbance of SOD was determined at 450 nm, MDA at 532 nm, GSH-Px at 340 nm, GSH at 412 nm, and CAT at 540 nm using a Biotek μQuant spectrofluorophotometer (Bio-Tek, Winooski, VT, USA).

### Analysis of Proinflammatory Markers

For serum enzyme-linked immunosorbent assay (ELISA), blood samples (3 ml) were collected from the abdominal aortic blood vessel and centrifuged (3000 r/min) for 10 min at 4 °C to separate the plasma from them. The supernatants thus obtained were frozen at − 80 °C for subsequent analysis, with the highly hemolytic samples discarded. For ELISA, the whole hippocampus and part of the cortex tissues were immediately collected into labeled tubes and stored at − 80 °C until analysis. Inflammatory markers in the serum, hippocampus and cortex were assayed by ELISA using commercial kits (rat interleukin-1beta (Cat. No. ERC007), interleukin-6 (ERC003) and tumor necrosis factor-alpha (ERC102a) ELISA Kit, Neobioscience Co., Ltd., Shenzhen, China). ELISA was performed with a Biotek Epoch spectrofluorophotometer (Bio-Tek, Winooski, VT, USA) following the manufacturer’s manual.

### Behavioral Assessment

Behavioral tests of the animals (n = 12/group) were performed from the second day after H group rats were transported back to Chengdu (500 m a.s.l.), China. The behavior test was conducted in a soundproofed and isolated room with weak illumination. The indoor temperature and ventilation were strictly controlled under the same conditions. All tests were performed between 9 am and 1 pm. The experimenter was blinded to the group division of the rats and the behavioral data analysis.

#### Open Field Test (OFT)

The OFT was conducted in a white plexiglass square arena (120 cm × 120 cm × 40 cm) with a black floor. The rats were placed at a stationary starting point in the center of the arena and allowed to freely explore the field for 15 min. The moving traces of the animals were videotaped using an overhead camera and analyzed by a computerized analysis system. The central field, by definition, is the central area of an open space of 40 × 40 cm^2^, which accounts for one quarter of the total area. After each test, the surface and sidewalls of the apparatus were thoroughly washed with 75% ethanol solution to eliminate any olfactory cues that might affect subsequent training results. The parameters measured included the duration of the travels in the central area (percentage of the total time) to evaluate anxiety-like behaviors and the total distance of the travels as a test of locomotive activity of the rats.

#### Elevated Plus Maze (EPM) Test

The apparatus of the EPM test was made of a black plexiglass, consisting of two opposing open arms (50 cm × 10 cm) and two closed arms (50 cm × 10 cm) that extended from a shared central platform (10 cm × 10 cm) at 90° shared by the arms. EPM was elevated by 50 cm above the floor. The rats were placed on the central platform with their head facing one open arm and were allowed to explore freely on the platform for 5 min. A video camera mounted on top of the maze was used to videotape the rats. Their movements were analyzed using a computerized analysis system. The number of open arm entries and the time spent in the open arms (% of total) were recorded to evaluate the general level of anxiety. During the intervals between the tests, the apparatus was carefully cleaned with 75% ethanol solution to remove prior odors.

#### Morris Water Maze (MWM) Test

The MWM is a task to evaluate the spatial learning and reference memory functions of rats, according to the procedures of Charles V. Vorhees [26]. It was conducted in a black circular pool (200-cm diameter × 60-cm height) with black-innocuous ink water (depth: 40 cm, temperature: 25 ± 1 °C) midway. A 10-cm diameter-colored target platform was placed in the fourth quadrant. The swimming tracks of the rats were recorded via a digital camera above the pool. The detailed protocol for cued learning, training and probe trials is described as follows.

(1) Cued learning test. On the first day, the swimming ability and visual state of rats in the two groups were detected. The platform was raised approximately 1.5–2 cm above the water surface. The rats entered the water from any random quadrant to find the platform and then rested on the platform for 30 s. (2) Training test. The rats were randomly put into the pool facing the wall at four water entry points, including the northeast, northwest, southeast, and southwest. In each experiment, each rat spent 60 s at most finding the hidden platform 1.5–2 cm beneath the water surface. Once the rats found the platform, they were allowed to sit on it for 10 s. The time the rats took from entering the water to finding the platform and climbing onto the platform was recorded as the escape latency. If the rats failed to climb onto the platform within the given time, the time was recorded as 60 s. After that, the rats were guided to stay on the platform for 15 s. Following this protocol, each animal was trained four times per day for 5 consecutive days. (3) Probe test. The platform was removed after the training test (on the 7th day), and then the animals were put into the water from the opposite quadrant to trace their movement trajectory for 60 s. The time, distance, speed of swimming and number of crossings of the original platform position in the target quadrant (the quadrant where the platform was originally placed) were recorded.

### MRI Acquisition

MRI scanning was conducted after the behavior assessment (n = 12/group). The small animal MRI examination was performed in a 7.0 T magnetic resonance imaging system (BioSpec 70/30; Bruker, Karlsruhe, Germany). For the animal study, a 72-mm open birdcage quadrature volume resonator was utilized for excitation, and a four-channel (2 × 2) phased array coil was used as a receiver. Anesthesia was initially induced with 2.5% isoflurane and was maintained with 1% to 1.5% isoflurane (2 L/min oxygen flow) during MRI scanning. The rats were placed in a prone position with their head fixed using a tooth rod and two ear pins. A respiratory rhythm sensor was installed beneath the abdomen to observe and monitor respiration at 50–80 breaths per minute. Additionally, the rats were kept on an animal heating pad connected to hot circulating water at 37 ± 1.0 °C during scanning to prevent the body temperature from falling. Multiparametric MR image scanning (including high-resolution T2, CEST, T1 map and DCE) was sequentially performed on each rat.

For VBM, high-resolution anatomical MRI data were captured using a T2-weighed turbo-rapid acquisition with relaxation enhancement (RARE) sequence. T2-weighted images (T2WI) were acquired using a two-dimensional RARE sequence with parameters including repetition time (TR) = 11,400 ms, echo time (TE) = 48 ms, number of averages = 6, slice thickness = 0.35 mm, slice gap = 0 mm, field of view (FOV) = 35 × 35 mm^2^, in-plane resolution of 0.137 mm^2^ × 0.137 mm^2^ (matrix size = 256 × 256), and total scanning time for T2WI of approximately 38 min.

When the high-resolution T2-weighted images of each rat scanning were obtained, other sequence scans were performed. For CEST, the B_0_ field over the whole rat brain was corrected using field mapping following higher-order shimming procedures. Continuous-wave CEST data were acquired using a fat-suppressed RARE sequence with an RF saturation pulse (TR = 5 s, TE = 30.73 ms, RARE factor = 24, matrix size = 96 × 96, slice thickness = 1.5 mm, FOV = 30 × 30 mm^2^). The RF saturation power (B1) and saturation length for CrCEST were 2.0 μT and 1 s, respectively [27]. The frequency measured to assess CrCEST ranged from 1 to 3.5 ppm at an interval of 0.05 ppm. The reference image (S_0_ image) was obtained by setting the offset at 200 ppm [28]. Quantitative T1 relaxation maps were acquired using RARE with variable repetition time sequences with seven TRs (0.5, 1, 1.5, 2, 3.5, 5, and 8 s), TE = 12 ms, RARE factor = 4. All CrCEST scans were selected from a single slice that was parallel to the coil and set to approximately − 2.36 mm (according to the results of VBM) in relation to the anterior commissure (AC).

For DCE–MRI, 120 continuous dynamic T1_FLASH images were acquired. The animals were manually intravenously given a bolus of gadopentetate dimeglumine as a contrast agent (0.2 mmol/kg, BeiLu Pharmaceutical Co., Ltd., Beijing, China) via the caudal vein after 10 to 15 baseline images (to cover 10 to 15 s of acquisition), and precontrast images were acquired. All DCE–MRI scans were selected as double slices that were parallel to the coil positioned and set to approximately − 2.36 mm and − 4.70 mm in relation to the AC. The scanning parameters included TR = 15.6 ms, TE = 1.8 ms, FOV = 35 × 35 mm^2^, flip angle of 12°, slice thickness of 0.8 mm and in-plane resolution of 0.273 × 0.273 mm^2^.

### MRI Data Processing

VBM: High-resolution T2-weighted imaging was performed using the statistical parametric mapping toolbox and software (SPM12, Wellcome Trust Centre for Neuroimaging, London, UK) in MATLAB 2013b. (1) Bet: High precise whole-brain masks for segmenting the nonbrain tissue for in vivo scans were achieved using PCNN3D [29], and the whole brain tissues were extracted with an image calculator in the SPM12 toolbox with the calculated approach to the rat’s unprocessed scans multiplied by their brain mask. An improper mask was embellished using ITK-SNAP [30]. (2) Origin setting and voxel size resizing: Each skull-stripped T2-weighted image was resized by a factor of 10 to account for variation in the whole-brain volume between humans and rodents [31]. Meanwhile, the resized T2-weighted images were manually reoriented to the origin. (3) Segmentation and spatial normalization: In the old segment batch in SPM12, the resized and reoriented T2-weighted images were segmented into tissue probability maps of the gray matter, white matter and cerebrospinal fluid with a unified segmentation approach [12]. Then, the tissue class images were nonlinearly normalized to the SIGMA template [32], and the signal intensities of the normalized maps were modulated by the Jacobian determinant to add the transformation information of regional or global volume generated by nonlinear transformations (such as warp and deform) in image segmentation results to account for voxelwise volume alteration in the cerebral regions [33] (Supplemental Fig. S1). (4) Smooth: The images were then smoothed with an 8-mm full-width-at-half-maximum Gaussian kernel for analysis. (5) Visualization of statistical analysis and results: After the obtained images were imported into the statistical analyzer in SPM12, contrast T-maps were generated and compared voxel by voxel with two-sample t tests with a gray matter mask settled at an absolute threshold mask of 0.2 (with relative total brain volumes entered as a covariate). The more stringent familywise error correction was used for multiple comparisons, with a statistical threshold of 0.05 and with a cluster size of 200 voxels. However, although image preprocessing was performed on the resized scales, the results of VBM analysis were still displayed on the original scales. The significantly altered clusters in the hippocampus were saved as masks, and their corresponding rGMV was extracted using the dpabi toolbox (http://rfmri.org/dpabi).

Rat CEST data processing was performed using custom-written MATLAB scripts (MathWorks, www.mathworks.com, version R2018b). Regions of interest (ROIs) were manually delineated on the left and right hippocampi of P and H group rats. The CrCEST peaks (*R*) in the *R*-spectrum were extracted using the polynomial and Lorentzian line‐shape fitting (PLOF) method as described previously [28, 34, 35]. Cr concentrations were quantified by the PLOF scheme based on the principle that the values of CrCEST peaks extracted from the Z spectrum are proportionate to the Cr concentration. CrCEST peaks can be represented by a Lorentzian function as follows:

1 $$R\, = \,R_{{{\text{exch}}}} \frac{{(w/2)^{2} }}{{(w/2)^{2} \, + \,(\Delta \, - \,\Delta _{{{\text{exch}}}} )^{2} }}$$

where w is the peak line width (full-width-at-half-maximum) of the Lorentzian line shape, $\Delta$ is the offset, Δ_exch_ is the chemical shift of the CrCEST peak (approximately 1.95 ppm in vivo) in relation to the water signal, and *R*_exch_ is the true apparent relaxation rate of the CEST peaks in the *R*- spectrum.

The broad background (*R*_back_) in the *R*‐spectrum can be represented by a mixed polynomial and Lorentzian function as follows:

2 $$R_{{{\text{back}}}} \, = \,\frac{{{\text{C}}0({\text{C}}1/2)^{2} }}{{({\text{C}}1/2)^{2} \, + \,\Delta \omega ^{2} }}\, + \,C2\, + \,C3 \cdot \Delta \omega$$

A broad background signal (*R*_back_), including direct water saturation (DS), magnetization transfer contrast (MTC), aromatic protons and other metabolites, was fitted by a higher-order polynomial function. The first Lorentzian function in Eq. (2) was used to account for DS, whereas C2 + C3∙Δω was used to fit MTC and other exchanging protons, such as amines and hydroxyls.

DCE: The Ktrans value was calculated using DCE@urLAB 1.0 software for Microsoft Windows 7 (Microsoft, Redmond, WA, USA; http://www.die.upm.es/im/archives/DCEurLAB/). All DCE images were analyzed as described previously [36]. To evaluate BBB permeability with DCE contrasts, two experienced observers blinded to each animal’s status manually delineated the borders of the ROIs in the hippocampus and the ipsilateral cortex of both hemispheres in slices 1 (AC, − 2.36 mm) and 2 (AC, − 4.70 mm), as shown in Supplemental Fig. S2, and prominently visible blood vessels were excluded based upon the T2-weighted images. Finally, the extended Tofts-Kety model including the parameter of the plasma volume was chosen to obtain the Ktrans values averaged for each rat. For each rat, the Ktrans value of the hippocampus or the cortex was calculated as a whole as the average of the Ktrans values of four ROIs of the two slices.

#### Histopathological Examination

Following MRI, the brains of all the animals were collected and underwent histological study. The animals were euthanized with an overdose of isoflurane and perfused transcardially with 250 ml of ice-cold saline, followed by 300 ml of 4% paraformaldehyde (PFA) in phosphate-buffered saline (PBS, 0.1 mol/L, pH 7.4) for 30 min at a rate of 12 mL/min. After perfusion, the brains were removed and fixed in 40 ml of 4% paraformaldehyde. After sufficient postfixation, the brains were embedded in paraffin. Four-micrometer-thick coronal sections were consecutively taken from approximately bregma − 2.36 mm by sledge microtome, which included the focus of the decreased rGMV values in the hippocampus observed in the H group compared with that in the P group.

##### Nissl Staining

The obtained tissue sections (six brains/group) were collected on glass slides, deparaffinized, stained with 1% cresyl violet solution by Nissl staining for morphological study of the hippocampus, and then examined under a light electric microscope (CM 3000; Leica, Bensheim, Germany).

##### Fluoro Jade-B Staining

For the neurodegeneration study, brain sections of the rats were stained with Fluoro Jade-B (FJB) dye, which is a fluorescent substance that binds sensitively and specifically to degenerating neurons [37]. Staining was performed according to the method of Schmued et al. [38]. After staining, degenerating neurons were counted under a fluorescence microscope (Nikon Eclipse C1, Tokyo, Japan). Images of CA1, CA3 and ventral dentate gyrus (DG) granule cells (upper lobe) were obtained at 40× magnification. The upper blade of DG granule cells showed neuronal susceptibility to hypoxia compared with the dorsal blade (lower blade) [39]. Two independent observers blinded to the group division of the rats used ‘Image-Pro Plus 6.0’ image analysis software (MediaCybernetics, Silver Spring, MD, USA.), manually counted the positive neurons of FJB in the hippocampal subregions of interest (CA1, CA3, and DG) per rat section with six rats per group (n = 6 rats/group) and each area in 3 to 5 sections.

##### Immunofluorescence Staining

Levels of antiglial fibrillary acidic protein (GFAP, 1:500, Wuhan Servicebio Biotechnology Co., Ltd., Wuhan, China), anti-ionized calcium binding adaptor molecule 1 (Iba-1, 1:1000, Ibid.) and antineuronal nuclei (1:1000, Ibid.) were used to detect astrocytes, microglia and neurons. After the paraffinized brain slides were dewaxed and rehydrated, microwave heat-induced antigens were retrieved with citrate (pH 9.0) for 15 min before they were incubated in 1 N hydrochloric acid (1 mol/L) for 20 min and fixed in 4% PFA for 10 min. Next, the sections were blocked with 3% BSA for 30 min at room temperature and incubated with a primary antibody in blocking serum at 4 °C overnight. The following day, the sections were incubated with secondary antibodies, including Alexa Fluor 488 goat anti-mouse and Cy3 goat anti-rabbit antibodies, for 1 h in the dark at room temperature. The cell nucleus was counterstained with 4,6-diamidino-2-phenylindole (DAPI) for 10 min in the dark. Each of the above sections was washed three times for 5 min with PBS. The slides were observed under an immunofluorescence microscope (Nikon Eclipse C1, Tokyo, Japan). GFAP-labeled and Iba1-labeled cells were counted in the hippocampal subregions of interest (CA1, CA3, and DG) per rat section for six rats per group (n = 6 rats/group), and each area was counted in 3–5 sections. The counting was performed by 2 independent observers blinded to the group division of the rats.

##### Golgi–Cox Staining

The modified Golgi–Cox staining method was used to detect the dendritic spines of hippocampal neurons as described previously [40]. Intracardial perfusion was performed with 500 ml of 0.9% saline at room temperature in rats deeply anesthetized with isoflurane. Immediately after perfusion, the brains were fixed in bottles of 50 ml filled with 40 ml of 4% PFA for 24 h. Then, the brains were impregnated, sectioned coronally (100 μm) containing the hippocampus and the cortex, and stained according to the instructions in the FD Rapid Golgi Stain Kit. Dendrite spine density and dendrite complexity were assayed using ImageJ software as described previously [41]. Dendrite spine densities under a 100× oil objective lens were counted by the number of spines along 30–90 µm segments of the second- or third-order dendritic branch arbitrarily selected in apical dendrites in hippocampal and cortical neurons (spine density = number of dendritic spines/length of dendrites × 10). Sholl analysis under a 20× oil objective lens was performed to evaluate the dendrite complexity by counting the number of intersections between the dendrites with an overlaid concentric grid at an interval of 10 μm. Three neuronal cells per brain slice and three brain slices per animal (n = 6 rats/group) were chosen for quantitative analysis. An observer blinded to the group division of the rats analyzed the number of dendritic spines with ImageJ (National Institutes of Health, Bethesda, MD, USA).

##### TdT-Mediated dUTP Nick-End Labeling (TUNEL) Assay

Apoptosis was quantified to observe DNA fragments by TUNEL reaction. After being dewaxed and rehydrated, the paraffin-embedded slices were incubated with proteinase K for 30 min. After incubation, the sections were washed in 0.1 M PBS for 5 min and then incubated in a permeabilized working solution at room temperature for 20 min. The sections were slightly dried and then incubated with a mixture of terminal deoxynucleotidyl transferase enzyme and digoxigenin-tagged dUTP for 2 h. Subsequently, the cell nuclei were counterstained using DAPI, and the slices were finally mounted with anti-fade mounting medium. TUNEL-positive neurons were counted by two blinded experimenters in the hippocampal subregions of interest (CA1, CA3, and DG) per rat section for six rats per group (n = 6 rats/group) and each area in 3–5 sections (× 200).

#### Transmission Electron Microscopy (TEM)

The ultrastructural changes in the hippocampus (CA1, CA3, and DG) and the cortex were observed using TEM. The rats (n = 3 rats/group) were deeply anesthetized using isoflurane as mentioned above, and the brains were perfused with 2.5% glutaraldehyde perfusion solution via the heart. We used a blade to cut and harvest hippocampal and cortical tissue blocks quickly (within 1 mm^3^). The removed tissue was immediately fixed in 3% glutaraldehyde (buffered at pH 7.4) for 18–20 h and refixed in 1% osmium tetroxide for 2 h at 48 °C. Following dehydration, the hippocampus and the cortex were embedded in acetone and epoxy resin (Epon 812), and ultrathin sections of 50 nm were fixed on cuprum grids. After staining with 1% uranyl acetate and lead citrate at room temperature, the ultrastructure of hippocampal and cortical neurons was observed using a JEM-1400FLASH (JEOL Ltd., Tokyo, Japan) TEM.

#### Statistical Analysis

Statistical analysis was performed using Prism Version 8.0 software (GraphPad Software, La Jolla, CA, USA). All data are expressed as histograms of means ± SD values. The two groups were compared using unpaired two-tailed Student’s *t test* or a Mann–Whitney nonparametric test according to data normality test confirmed by quantile–quantile plot. For the MWM test and Sholl analysis, two-way repeated ANOVA followed by Sidak’s post hoc test was used. Pearson’s correlation coefficient (*r*) between behavioral parameters and MRI parameters was calculated. A value of *P* < 0.05 was considered to be statistically significant.

## Results

### Body Weight, Blood Routine and Arterial Pressure

After 8 months of exposure to plateau hypoxia, the body weight of the H group rats was higher than that of the P group rats (*P* < 0.05). Red blood cells, hemoglobin, white blood cells and granulocyte counts were significantly higher in the H group than in the P group (*P* < 0.05). The mean pulmonary arterial pressure of the H group was significantly higher than that of the P group (*P* < 0.05), whereas the mean systemic arterial pressure of the H group was not influenced by exposure to high-altitude hypoxia (*P* > 0.05) (Table 1), as we expected.

**Table 1:** Body weight, full blood count, and hemodynamic parameters of both groups

	H (n = 12)	P (n = 12)	*P* value
Weight			
Before plateau hypoxia (g)	99 ± 5	100 ± 3	0.134
After plateau hypoxia (g)	665 ± 63	620 ± 36	0.032
Hematological analysis			
WBC (million/mm^3^)	8.6 ± 2.1	4.7 ± 1.7	0.001
Lymphocyte (%)	41.9 ± 10.4	57.1 ± 19.2	0.061
Monocyte (%)	5.5 ± 3.2	4.9 ± 2.5	0.681
Granulocyte (%)	51.7 ± 9.1	36.5 ± 17.4	0.041
RBC (million/mm^3^)	11.6 ± 0.5	10.3 ± 0.4	< 0.001
MCV (%)	46.2 ± 9.5	46.9 ± 1.3	0.824
Hb (g/dl)	164.3 ± 9.2	135.4 ± 4.4	< 0.001
Hemodynamic parameters			
PAPm (mmHg)*	22.9 ± 5.2	15.8 ± 1.6	0.0013
SAPm (mmHg)*	97.2 ± 13.6	100.8 ± 18.4	0.6818

Values are expressed as the mean ± SD. Lymphocytes, monocytes and granulocytes expressed in %

*WBC* white blood cells (million/mm^3^), *RBC* red blood cells (million/mm^3^), *MCV* mean cell volume (% volume), *Hb* hemoglobin (g/dl), *PAPm* mean pulmonary arterial pressure, *SAPm* mean systemic arterial pressure, *H* plateau hypoxia, *P* plain

*n = 8 per group

### Oxidative Stress Parameters in the Hippocampus and the Cortex

The levels of MDA in the hippocampal and cortical tissues of the H group were significantly higher than those of the P group (*P* < 0.05). The activities of GSH and antioxidant enzymes (SOD, CAT, GSH-Px) in the hippocampal and cortical tissues of H group rats decreased significantly compared with that of P group rats (*P* < 0.05) (Table 2).

**Table 2:** Effect of chronic exposure to a plateau hypoxic environment on oxidative stress parameters in the hippocampus and the cortex of H and P group rats

	Hippocampus			Cortex		
	H	P	*P* value	H	P	*P* value
MDA (nmol/mg prot)	0.13 ± 0.02	0.10 ± 0.01	0.033	0.15 ± 0.02	0.11 ± 0.01	0.019
SOD (U/mg prot)	0.86 ± 0.13	1.10 ± 0.06	0.026	0.49 ± 0.09	0.85 ± 0.16	0.013
CAT (nmol/min/mg)	79.24 ± 17.19	105.40 ± 5.27	0.045	80.34 ± 4.90	126.77 ± 9.51	< 0.001
GSH (μg/mg)	20.92 ± 2.28	26.30 ± 1.89	0.037	28.49 ± 4.51	45.15 ± 7.47	0.016
GSH-PX (U/mg prot)	0.020 ± 0.004	0.032 ± 0.002	0.006	0.022 ± 0.004	0.032 ± 0.006	0.043

Values are expressed as the mean ± SD (n = 6 per group)

*MDA* malondialdehyde, *GSH* glutathione, *SOD* superoxide dismutase, *CAT* catalase, *GSH-Px* glutathione peroxidase, *H* plateau hypoxia, *P* plain

### Proinflammatory Cytokine Levels in the Blood, Hippocampus, and Cortex

**Fig. 2:**
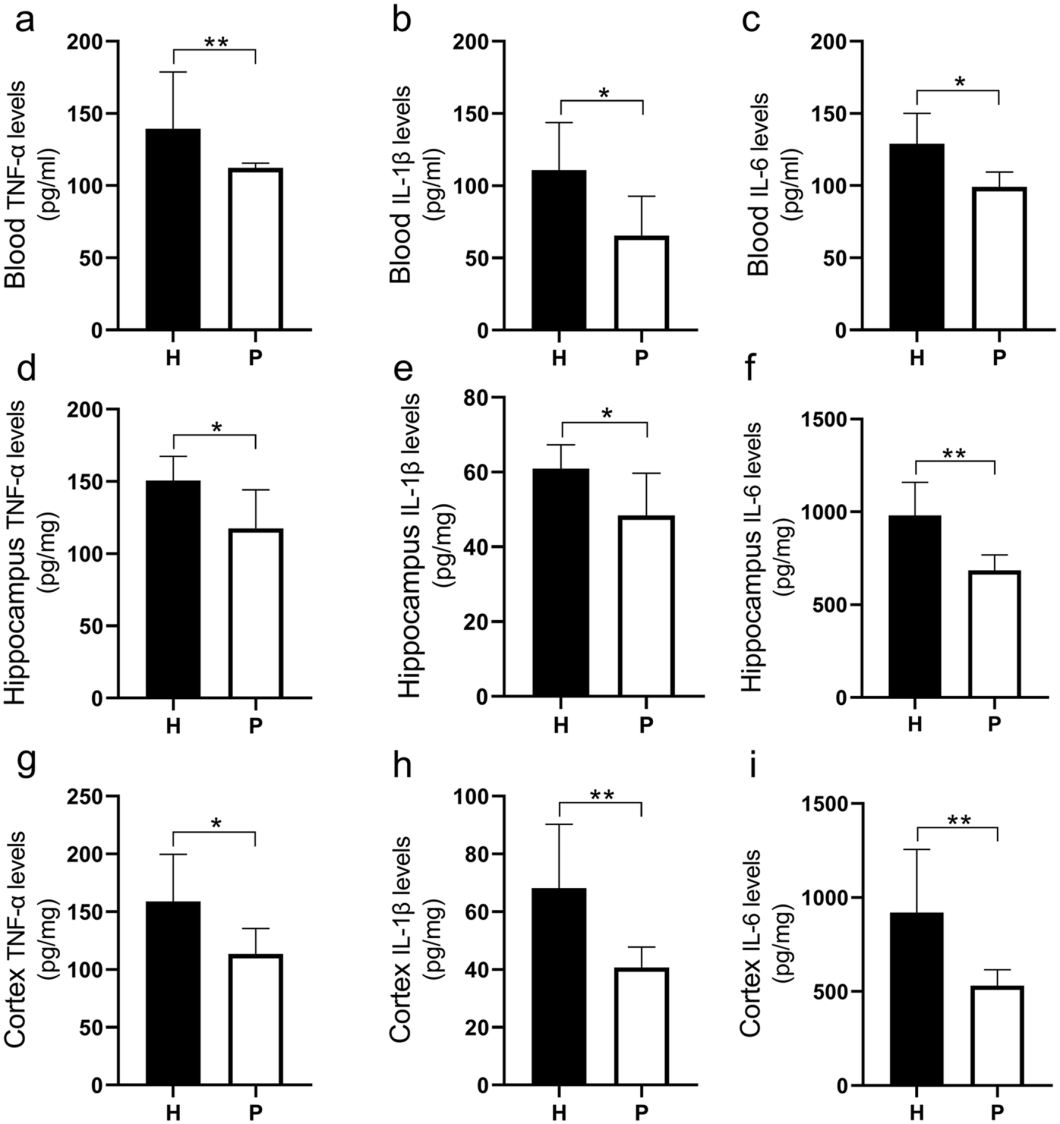
Bar graphs show between-group differences in TNF-α (**a**, **d**, and **g**), IL-1β (**b**, **e**, and **h**), and IL-6 (**c**, **f**, and **i**) in the blood, hippocampus and cortex tissue. Data are expressed as the mean ± SD (n = 6 per group). *H* plateau hypoxia, *P* plain, *TNF-α* tumor necrosis factor-alpha, *IL-1β* interleukin-1beta, *IL-6* interleukin-6. *P < 0.05, **P < 0.01

ELISA results in Fig. 2a–i show that, compared with that of the P group, the levels of tumor necrosis factor-alpha, interleukin-6, interleukin-1beta in the blood, hippocampus and cortex of the H group increased significantly (*P* < 0.05).

### Behavioral Test Results

#### Plateau Hypoxia Exposure Induced Anxiety-Related Behaviors

**Fig. 3:**
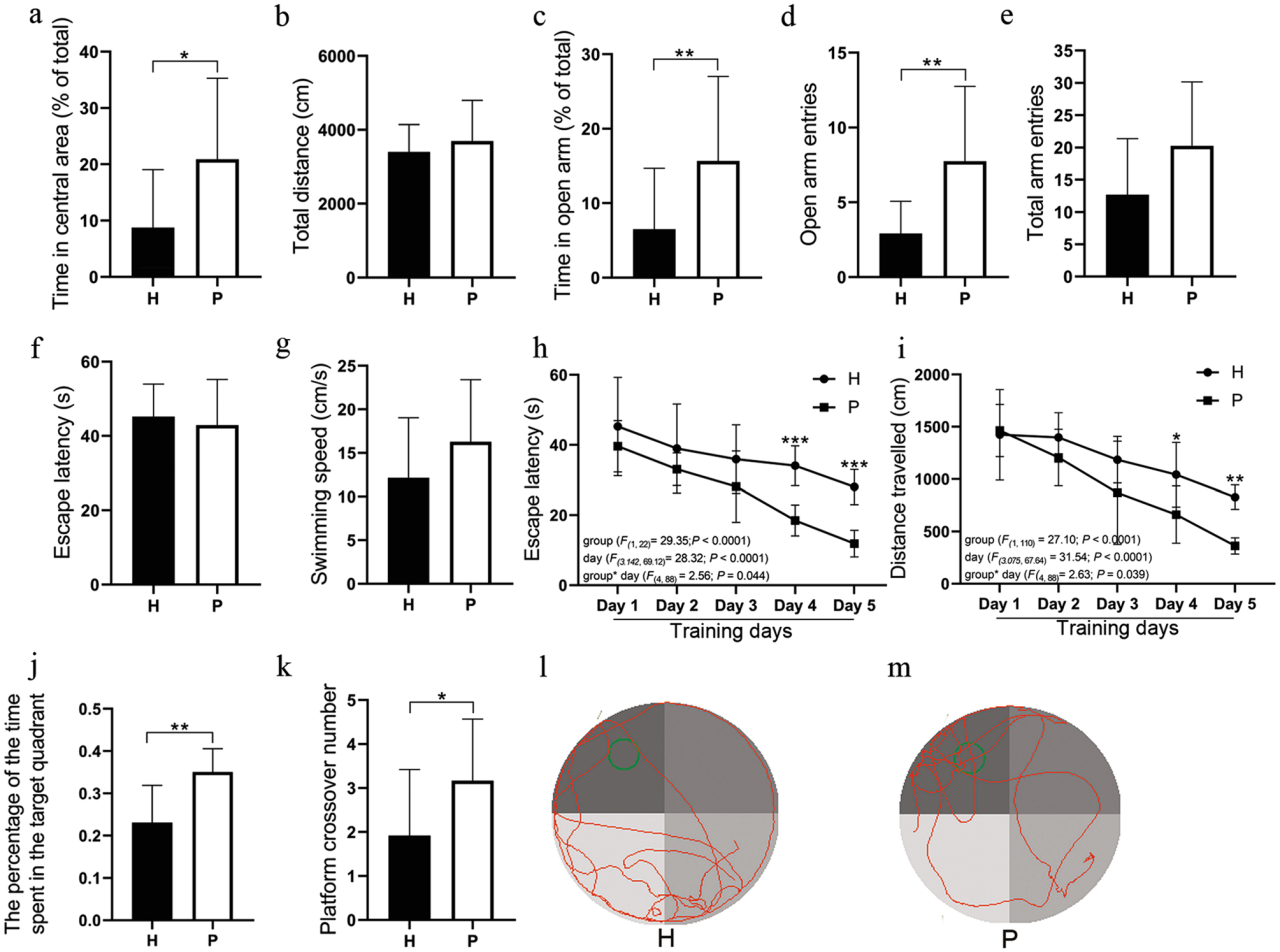
Between-group differences in the results of the OFT (**a**, **b**), EPM (**c**–**e**), and MWM (**f**–**m**). In the OFT, time in the central area (% of total) (**a**) and total distance traveled (**b**) were obtained. In the EPM test, time in open arms (% of total) (**c**), open arms entries (**d**), and total arm entries (**e**) were obtained. In the visible platform trial of the MWM, escape latency (**f**) and swimming speed (**f**) to the platform over water in the target quadrant were obtained. In the hidden platform trial of the MWM, the escape latency (**h**) and distance traveled (**i**) to the underwater platform in the target quadrant were obtained during the 2–6 day training phase. The times of crossing the original platform location (**j**) and time spent in the target quadrant (% of total) (**k**) were obtained during the probe trial of the MWM on the 7th day. Representative swimming paths of rats during the probe trial on Day 7 are depicted (**l** and **m**). Data are expressed as the mean ± SD (n = 12 per group). *H* plateau hypoxia, *P* plain, *OFT* open field test, *EPM* elevated plus maze, *MWM* Morris water maze. *P < 0.05, **P < 0.01, ***P < 0.001

As shown in Fig. 3, compared with the P group rats, the H group rats spent less time in the central square of the OFT, whereas the total distance traveled by the H group rats remained unaltered (Fig. 3a and b). EPM results showed that H group rats spent less time in and had fewer entries into the open arms, whereas the total number of arm entries remained unaltered (Fig. 3c–e). All these data suggest that plateau hypoxia exposure could induce anxiety-like behaviors in rats.

#### Plateau Hypoxia Exposure Induced Reduced Spatial Memory

In the visible platform trial, no significant differences in escape latency (H group rats: 45.22 ± 8.40 vs. P group rats: 42.93 ± 11.80, *P* > 0.05) or swimming speed (H group rats: 12.16 ± 6.59 vs. P group rats: 16.29 ± 6.82, *P* > 0.05) were found between the two groups (Fig. 3f and g). Repeated measurements with ANOVA for the escape latency and traveled distance to find the hidden platform across the training days indicate a significant effect on the groups [*F*(1, 22) = 29.35; *P* < 0.0001 for the escape latency and *F*(1, 22) = 27.10; *P* < 0.0001 for the traveled distance] and training days [*F*(3.142, 69.12) = 28.32; *P* < 0.0001 for the escape latency and *F*(3.075, 67.64) = 31.54; *P* < 0.0001 for the traveled distance], with a significant interactive effect of the two [*F*(4, 88) = 2.56; *P* = 0.044 for the escape latency and *F*(4, 88) = 2.63; *P* = 0.039 for the traveled distance]. Sidak’s multiple comparisons revealed that the escape latency and distance traveled to reach the platform significantly increased in the H group rats compared with the P group rats on the 4th and 5th days of the learning trials (Fig. 3h and i). Probe trials were conducted to assess spatial memory performance at 24 h after the 5th day of training. H group rats spent less time percentage than P group rats in the target quadrant of the pool, in which the hidden platform had been located beforehand (H group rats: 23.11 ± 2.54% vs. P group rats: 35.04 ± 1.59%, *P* < 0.01) (Fig. 3j) and H group rats made fewer platform crossings than P group rats did (H group rats: 1.92 ± 1.44 vs. P group rats: 3.17 ± 1.34, *P* < 0.05) (Fig. 3k). All these data suggest that plateau hypoxia exposure decreased the spatial memory performance of H group rats in comparison with that of P group rats.

### Multimodal MR Imaging Findings

#### Whole-Brain VBM

**Fig. 4:**
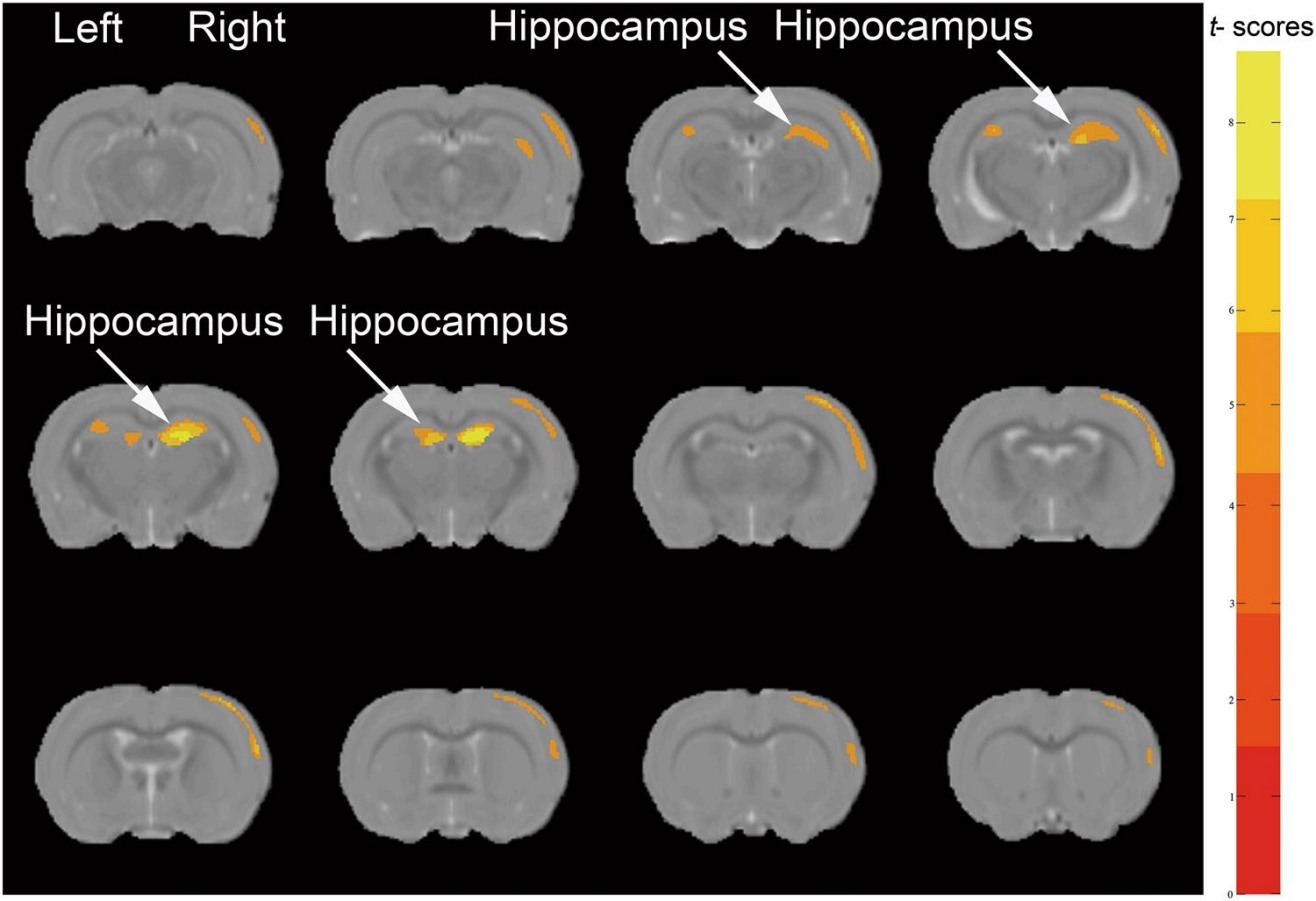
Whole-brain voxel-based morphometry analysis results. Red-to-yellow colors represent decreased gray matter volume after plateau hypoxia exposure (n = 12 per group; familywise error-corrected *p* < 0.05). Color bar units refer to t-scores

As expected, H group rats exhibited decreased rGMV (*P* < 0.05, familywise error-corrected) in their hippocampus compared with P group rats (Table 3; Fig. 4), which was also found in the primary somatosensory cortex of H group rats (Ibid.).

**Table 3:** The peak of the local maxima within each significant cluster (*P* < 0.05, familywise error-corrected) showed significant regional gray matter volume decreases in H group rats compared with those in P group rats

Brain regions	Coordinates (x, y, z)	Peak intensity	Z score	Voxels
Hippocampus (L)	(− 0.6, 3.59, − 2.36)	− 8.6411	6.20	2710
Primary somatosensory cortex forelimb (R)	(1.5, 3.59, − 9.60)	− 6.8465	5.36	2842

The atlas coordinates of the peaks are relative to bregma in the medial–lateral (x), superior–inferior (y), and anterior–posterior (z) directions (mm)

*H* plateau hypoxia, *P* plain

#### CrCEST

**Fig. 5:**
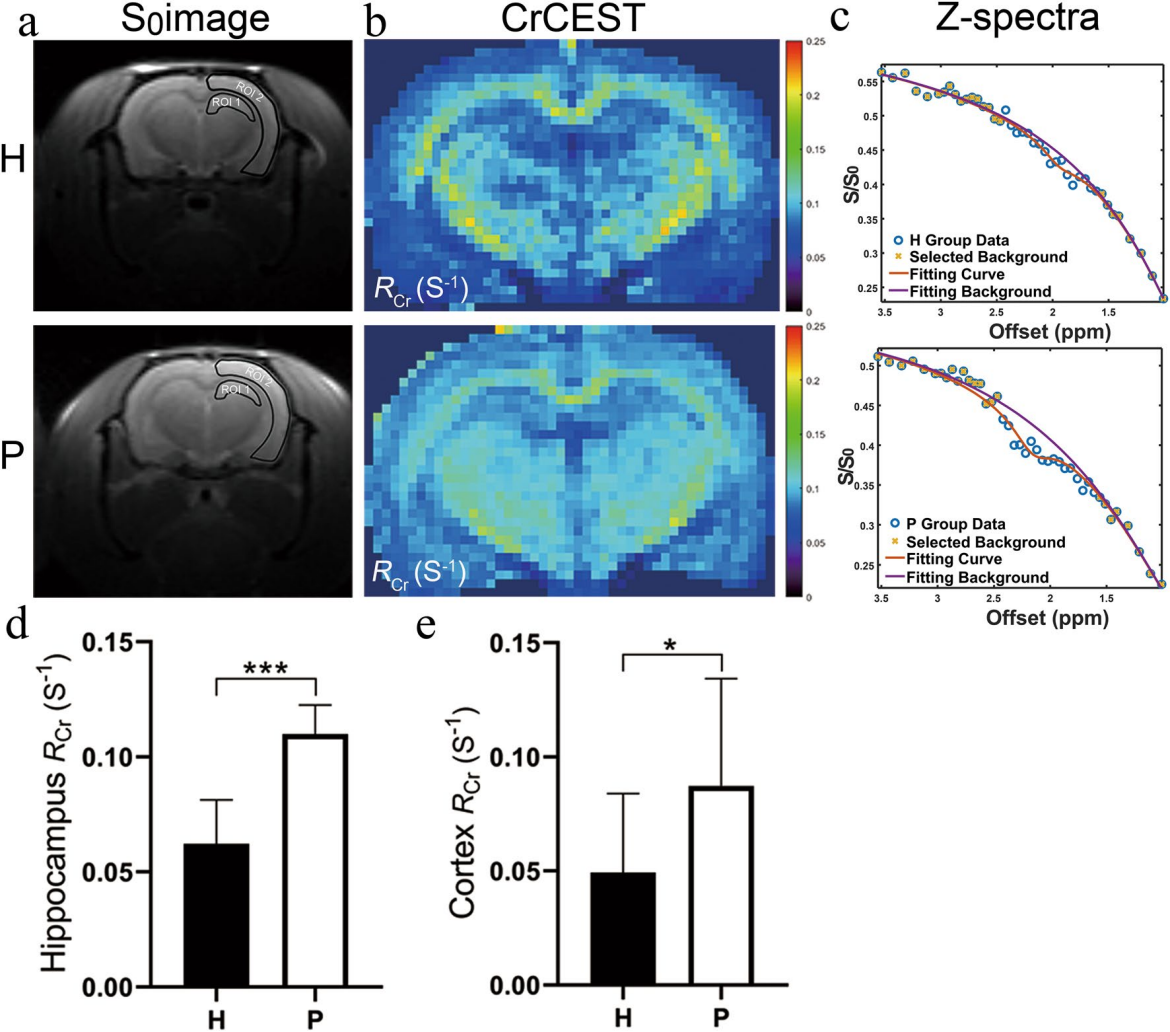
Representative CrCEST in the hippocampus and the cortex of H and P group rats. Typical S_0_ images (**a**), cortical CrCEST maps (**b**), CrCEST Z-spectra (**c**) for H and P group rats. Both CrCEST Z-spectra and CrCEST maps (*R*_Cr_) of H group rats show a clear signal reduction compared with P group rats. The ROIs used to extract regional values are indicated in (**a**). The Z-spectra in c are from the hippocampus (ROI 1). Data are expressed as the mean ± SD (n = 12 per group). *H* plateau hypoxia, *P* plain, *ROI* region of interest. *P < 0.05, ***P < 0.001. Bar graphs show differences in the CrCEST apparent relaxation rates in the rotating frame (*R*_*Cr*_) for hippocampus (**d**) and cortex (**e**) between the H and P group rats

Apparent relaxation rates of Cr (*R*_Cr_) maps of the brains of the two groups of rats as well as the corresponding S_0_ images are shown in Fig. 5a and b. Typical CrCEST Z-spectra of the hippocampus (black ROI 1) and the cortex (black ROI 2) in H and P group rats are plotted in Fig. 5c along with the PLOF fitting curves. We selected the averaged *R*_Cr_ to represent CrCEST data. *R*_Cr_ is linearly related to the Cr concentration, indicating that *R*_Cr_ is not susceptible to the scale-down effect induced by DS and MTC in CEST contrast imaging [28, 35]. *R*_Cr_ values in the hippocampus (0.062 ± 0.018 s^−1^ vs. 0.110 ± 0.012 s^−1^, *P* < 0.001) (Fig. 5d) and the cortex of H group rats (0.049 ± 0.033 s^−1^ vs. 0.087 ± 0.045 s^−1^, *P* < 0.05) (Fig. 5e) decreased significantly compared with that of P group rats.

#### DCE–MRI

**Fig. 6:**
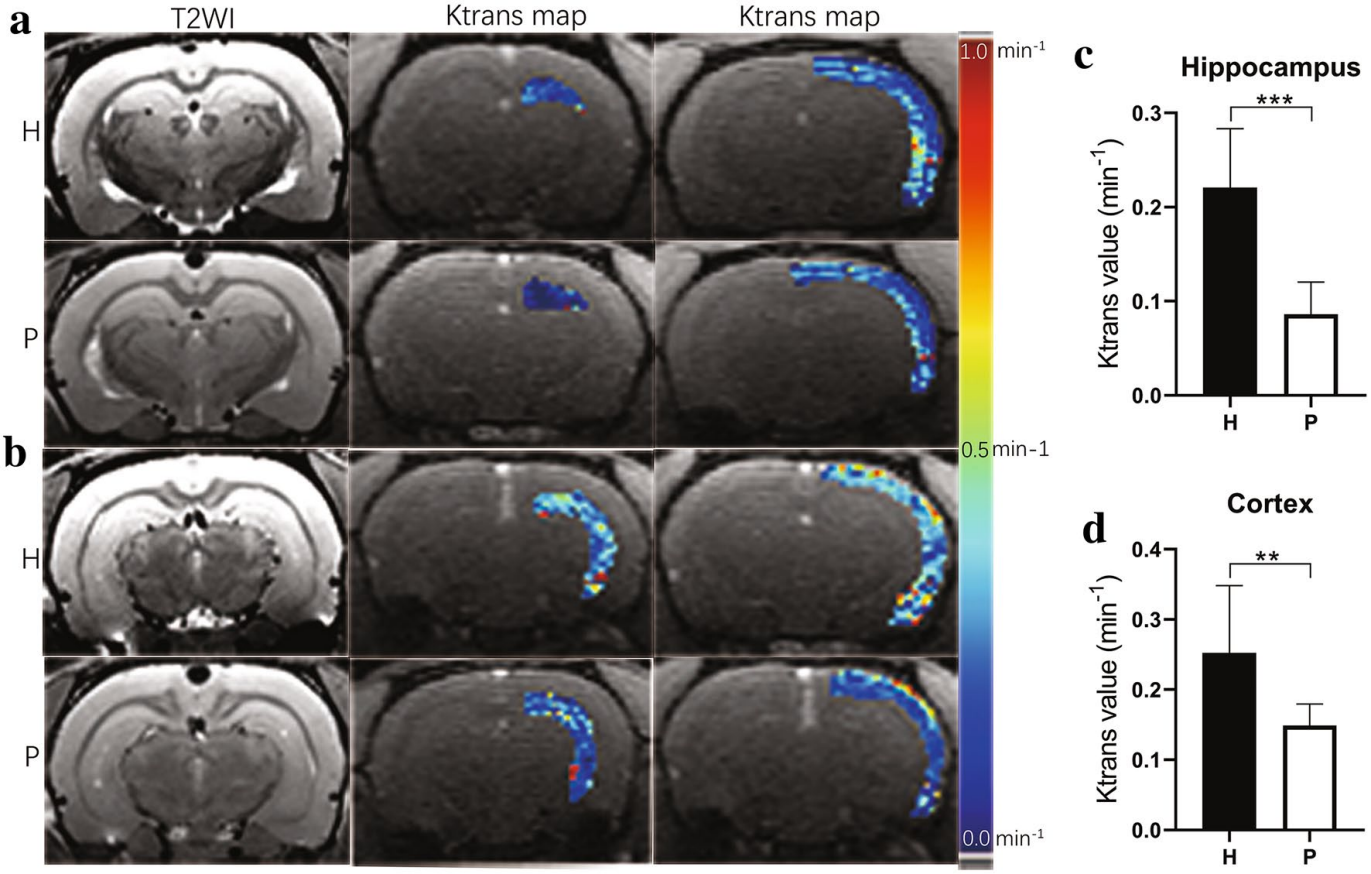
BBB permeability increase in the hippocampus and ipsilateral cortex of H group rats. **a** Hippocampus and cortex of H and P group rats at − 3.60 mm relative to the anterior commissure (slice 1). **b** Hippocampus and cortex of H and P group rats at − 5.60 mm relative to the anterior commissure (slice 2). Left column shows hippocampus and cortex on T2WI. Middle column shows Ktrans maps of hippocampus. Right column shows Ktrans maps of cortex. Color-coded Ktrans ranged between 0.0 and 1.0 min^−1^, with dark blue indicating 0.0 min^−1^, green indicating 0.5 min^−1^, and red indicating 1 min^−1^. **c** Ktrans of the right hippocampus of H and P group rats. **d** Ktrans of the right cortex of H and P group rats. Data are expressed as the mean ± SD (n = 12 per group). *H* plateau hypoxia, *P* plain, *BBB* blood–brain barrier, *Ktrans* volume transfer constant. **P < 0.01, ***P < 0.001

Quantitative analysis of Ktrans maps of H and P group rats is shown in Fig. 6a and b. Compared with P group rats, H group rats showed significantly higher Ktrans values in the hippocampus (0.221 ± 0.060 min^−1^ vs. 0.086 ± 0.033 min^−1^, *P* < 0.001) (Fig. 6c) and the cortex (0.253 ± 0.092 min^−1^ vs. 0.149 ± 0.030 min^−1^, *P* < 0.01) (Fig. 6d).

### Correlation Analysis

**Fig. 7:**
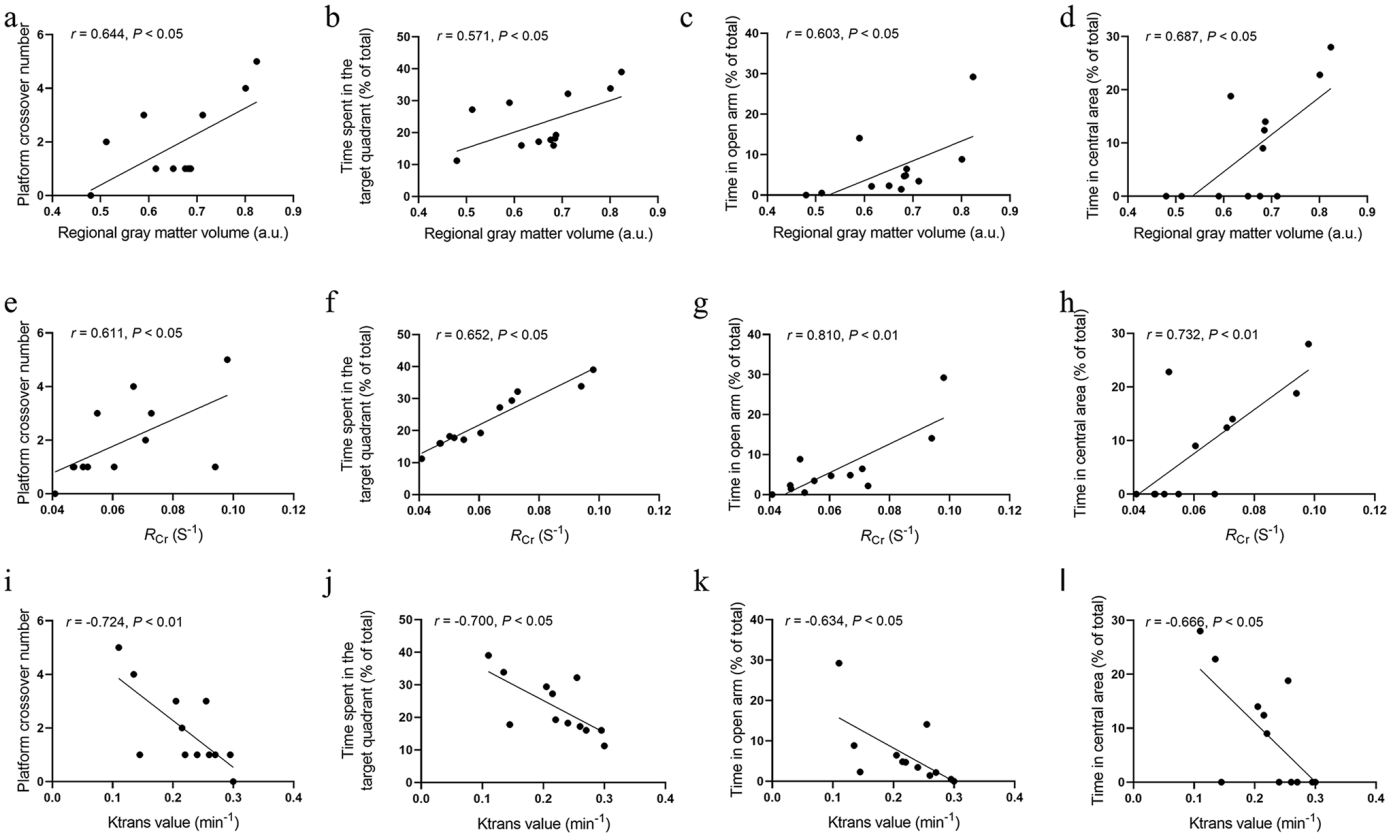
Correlation analysis results. Regional gray matter volume (rGMV) of the hippocampus was positively correlated with the platform crossover numbers (**a**), time spent in the target quadrant (% of total) (**b**), time in the open arm (**c**), and time in central area (% of total) (**d**). The *R*_Cr_ of the hippocampus was positively correlated with the platform crossover numbers (**e**), time spent in the target quadrant (% of total) (**f**), time in the open arm (**g**), and time in the central area (% of total) (**h**). Ktrans of the hippocampus was negatively correlated with the platform crossover numbers (**i**), time spent in the target quadrant (% of total) (**j**), time in the open arm (**k**), and time in the central area (% of total) (**l**)

As shown by the correlation analysis results in Fig. 7, rGMV of the hippocampus was positively correlated with the number of platform crossings (*r* = 0.644, *P* < 0.05), time spent in the target quadrant (% of total) (*r* = 0.571, *P* < 0.05), time spent in the open arm (*r* = 0.603, *P* < 0.05) and time spent in the central area (% of total) (*r* = 0.687, *P* < 0.05). The *R*_Cr_ of the hippocampus was positively correlated with the number of platform crossings (*r* = 0.611, *P* < 0.05), time spent in the target quadrant (% of total) (*r* = 0.652, *P* < 0.05), time spent in the open arm (*r* = 0.810, *P* < 0.01) and time spent in the central area (% of total) (*r* = 0.732, *P* < 0.01). Ktrans of the hippocampus was negatively correlated with the number of platform crossings (*r* = − 0.724, *P* < 0.01), time spent in the target quadrant (% of total) (*r* = − 0.700, *P* < 0.05), time spent in the open arm (*r* = − 0.634, *P* < 0.05) and time spent in the central area (% of total) (*r* = − 0.666, *P* < 0.05).

### Nissl Staining

**Fig. 8:**
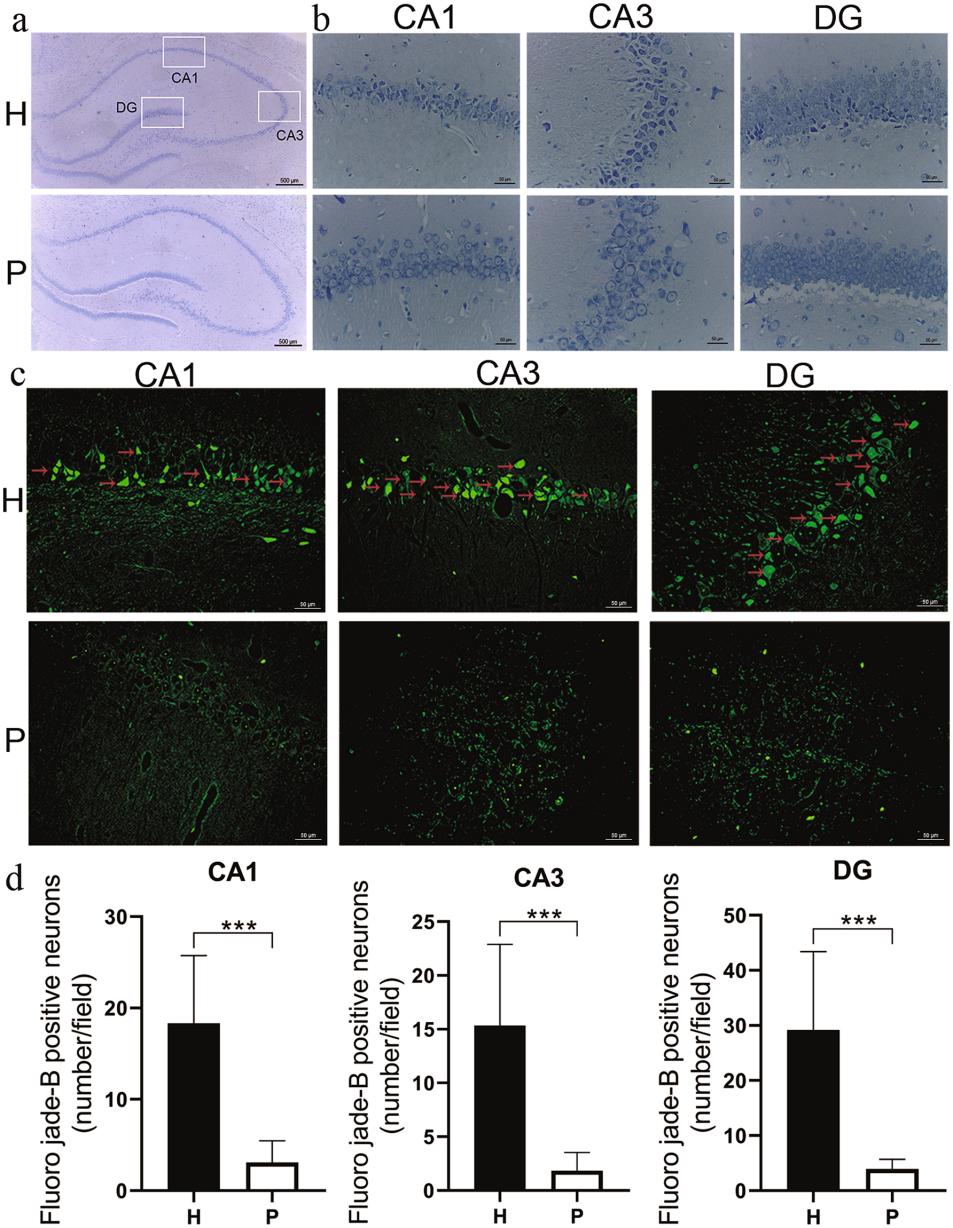
Effect of plateau hypoxia exposure on morphological changes in hippocampal neurons of rats. Representative scheme of the CA1, CA3, and DG subregions considered in the analysis (**a**). In the hippocampus of H group rats, pyknotic, small sized, dense and irregularly shaped cells were observed by Nissl staining (**b**). Degenerative neurons (red arrow) were labeled by Fluoro Jade-B staining (**c**). The graph represents the morphometric results of Fluoro Jade-B staining (**d**). Scale bar = 500 μm in (**a**) and 50 μm in (**b**) and (**c**). Data are expressed as the mean ± SD (n = 6 per group). *H* plateau hypoxia, *P* plain. ***P < 0.001

H group rats showed significant morphological changes in their neurons in the CA1, CA3 and DG areas of the hippocampus. After 8 months of plateau hypoxia exposure, scattered, small, irregularly shaped and shrinking neurons and nuclear pyknosis were observed in the CA1, CA3 and DG areas of the hippocampus of H group rats (Fig. 8b).

### FJB-Staining

FJB-staining revealed neurodegeneration of the hippocampus in the H and P group rats (Fig. 8c). H group rats showed a significant increase in FJB-positive cells in the CA1, CA3, and DG regions of the hippocampus compared with P group rats (*P* < 0.001) (Fig. 8d).

### Immunofluorescent Examination of Iba-1

**Fig. 9:**
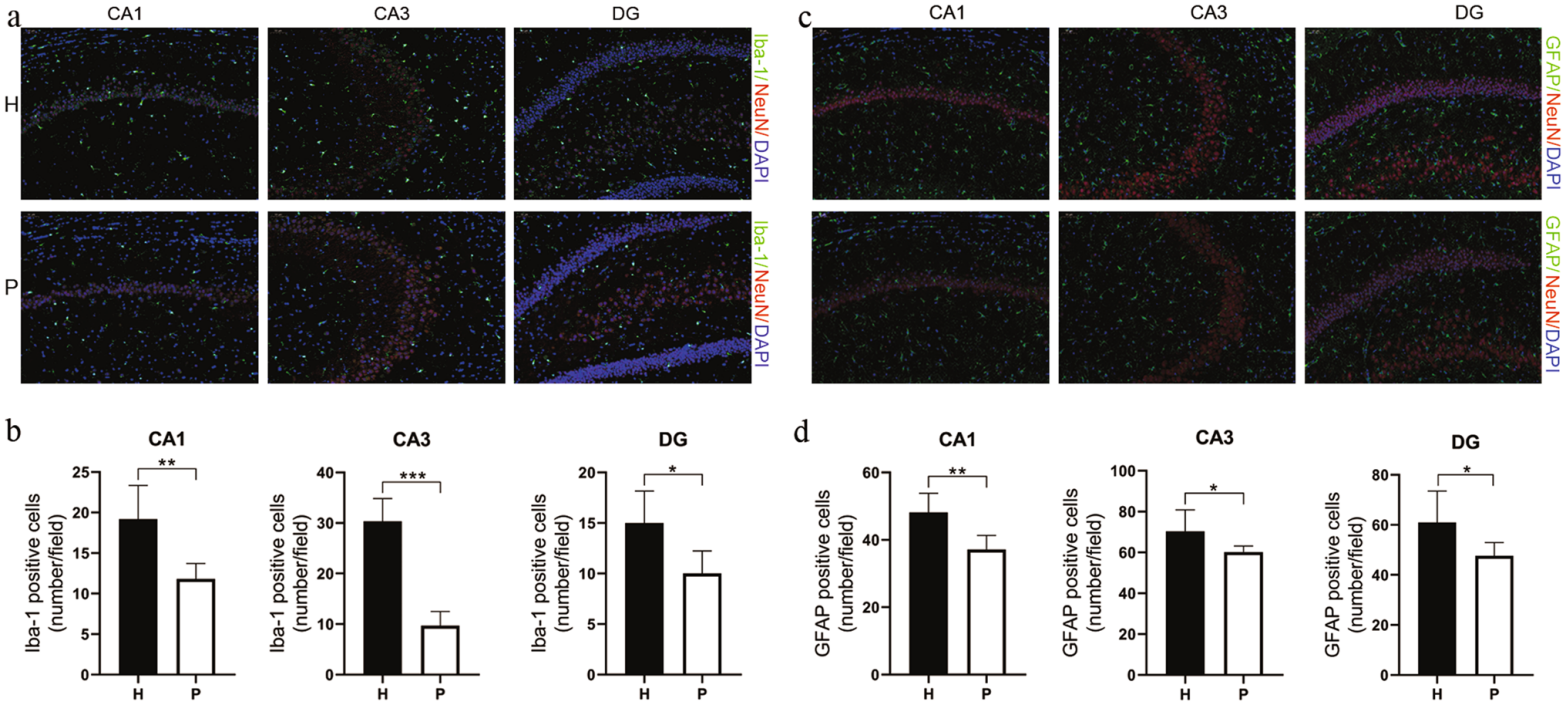
Iba-1 and GFAP increase in the hippocampus of H group rats. Representative immunofluorescence staining of microglia (Iba-1-positive cells) in the CA1, CA3, and DG areas (**a**). Bar graphs showing Iba-1-positive cell counts in the CA1, CA3, and DG areas (**b**). Representative immunofluorescence staining of astrocytes (GFAP-positive cells) in the CA1, CA3, and DG areas (**c**). Bar graphs showing GFAP-positive cell counts in the CA1, CA3, and DG areas (**d**). Data are expressed as the mean ± SD (n = 6 per group). *H* plateau hypoxia, *P* plain, *NeuN* neuronal nuclei, *Iba-1* ionized calcium binding adaptor molecule-1, and *GFAP* glial fibrillary acidic protein. *P < 0.05, **P < 0.01, ***P < 0.001

H group rats, compared with P group rats, showed a significant increase in their microglial cell counts in the CA1 (19.20 ± 3.71 vs. 11.80 ± 1.72, *P* = 0.0068), CA3 (30.33 ± 4.11 vs. 9.67 ± 2.56, *P* < 0.001) and DG (15.00 ± 2.83 vs. 10.00 ± 2.00, *P* = 0.0203) regions of the hippocampus (Fig. 9a and b).

#### Immunofluorescent Examination of GFAP

Compared with the P group rats, the H group rats showed a significant increase in astrocyte cell counts in the CA1 (48.17 ± 5.15 vs. 37.17 ± 3.80, *P* = 0.0032), CA3 (70.33 ± 9.66 vs. 60.17 ± 2.67, *P* = 0.0476) and DG (61.00 ± 11.43 vs. 47.67 ± 4.85, *P* = 0.0373) regions of the hippocampus (Fig. 9c and d).

#### Hippocampal Dendritic Complexity and Dendritic Spine Densities

**Fig. 10:**
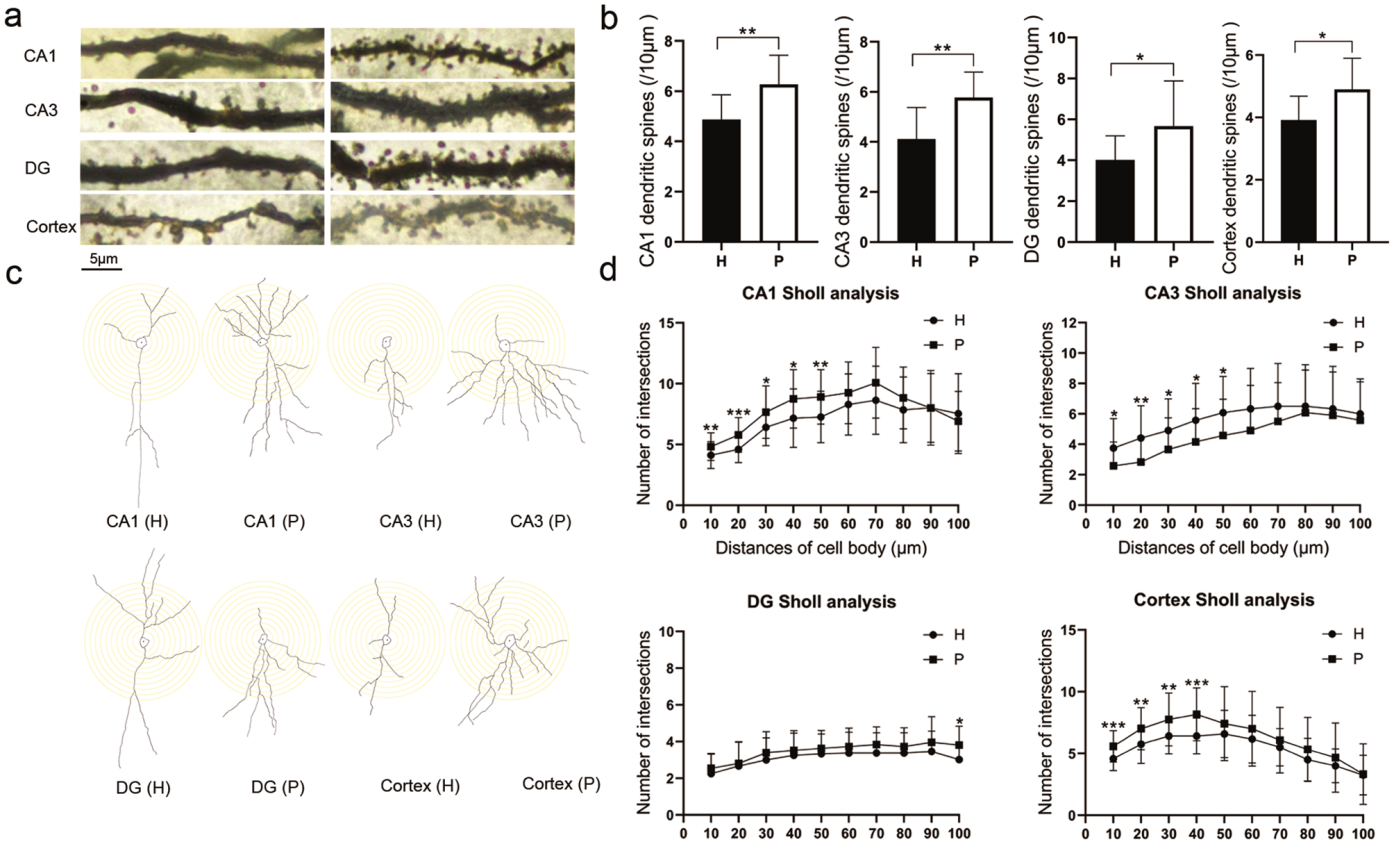
Modified Golgi–Cox staining showing the differences in hippocampal and cortical neurons between the H and P group rats. Neurons of H and P group rats were imaged under a 100 × lens (**a**). The H group rats showed a reduced number of dendrites compared with the P group rats (**b**). Schematic photomicrographs of neurons (20×) with the allocation of dendrites between repeated 10 μm-spaced concentric rings (**c**). The H group rats showed a reduced number of neuronal intersections in the hippocampus and cortex compared with the P group rats (**d**). Scale bar = 5 μm. Data are expressed as the mean ± SD (n = 6 per group). *H* plateau hypoxia, *P* plain. *P < 0.05, **P < 0.01

Compared with the control rats, the H group rats showed a decrease in the number of dendritic spines in the hippocampal CA1, CA3, and DG subregions and in the cortical region (*P* < 0.05) (Fig. 10a and b). The Sholl plots showed that dendritic intersections were distributed at an increasing distance from the center of the cell body (Fig. 10c). Repeated measurements with ANOVA of the number of intersections across the distances from the center of the cell body indicate a significant effect for the group [*F*(1, 94) = 7.218; *P* = 0.009 in the CA1 region, *F*(1, 94) = 5.671; *P* = 0.019 in the CA3 region, *F*(1, 94) = 5.350; *P* = 0.023 in the DG region, and *F*(1, 94) = 9.931; *P* = 0.002 in the cortical region], with a significant interaction between group and distance from the cell soma [*F*(9, 846) = 4.151; *P* < 0.0001 in the CA1 region, *F*(9, 846) = 4.148; *P* < 0.0001 in the CA3 region, *F*(9, 846) = 1.205; *P* = 0.029 in the DG region, and *F*(9, 846) = 2.068; *P* = 0.030 in the cortical region]. Sidak’s multiple comparisons show that compared with the control P group rats, H group rats had fewer Sholl intersections within 10–50 µm from the soma in the CA1 region, fewer Sholl intersections within 10–50 µm from the soma in the CA3 region, fewer Sholl intersections in 100 µm from the soma in the DG region, and fewer Sholl intersections within 10–40 µm from the soma in the cortex regions (Fig. 10d).

#### TUNEL Stain

**Fig. 11:**
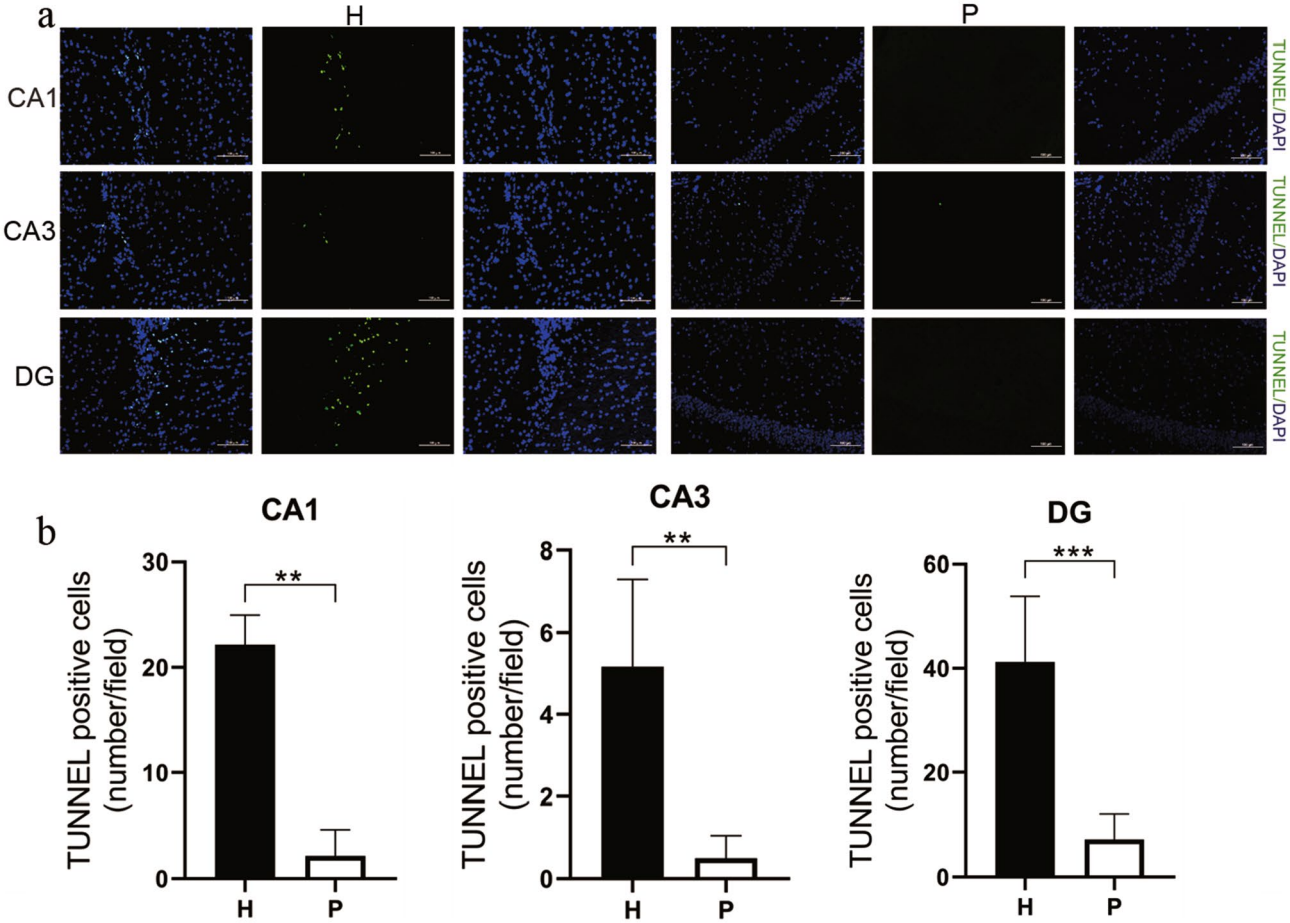
TUNEL immunofluorescent staining in the CA1, CA3, and DG areas (**a**). Bar graphs showing TUNEL-positive cell counts in the CA1, CA3, and DG areas. Scale bar = 100 μm. Data are expressed as the mean ± SD (n = 6 per group). *H* plateau hypoxia, *P* plain, *TUNEL* TdT-mediated dUTP Nick-end Labeling. ***P < 0.001

TUNEL-positive cells were observed in the hippocampal CA1, CA3 and DG subregions (Fig. 11a). A considerable number of TUNEL-positive neurons appeared in the hippocampal CA1, CA3, and DG of H group rats compared with that of P group rats (*P* < 0.01) (Fig. 11b).

#### TEM Results

**Fig. 12:**
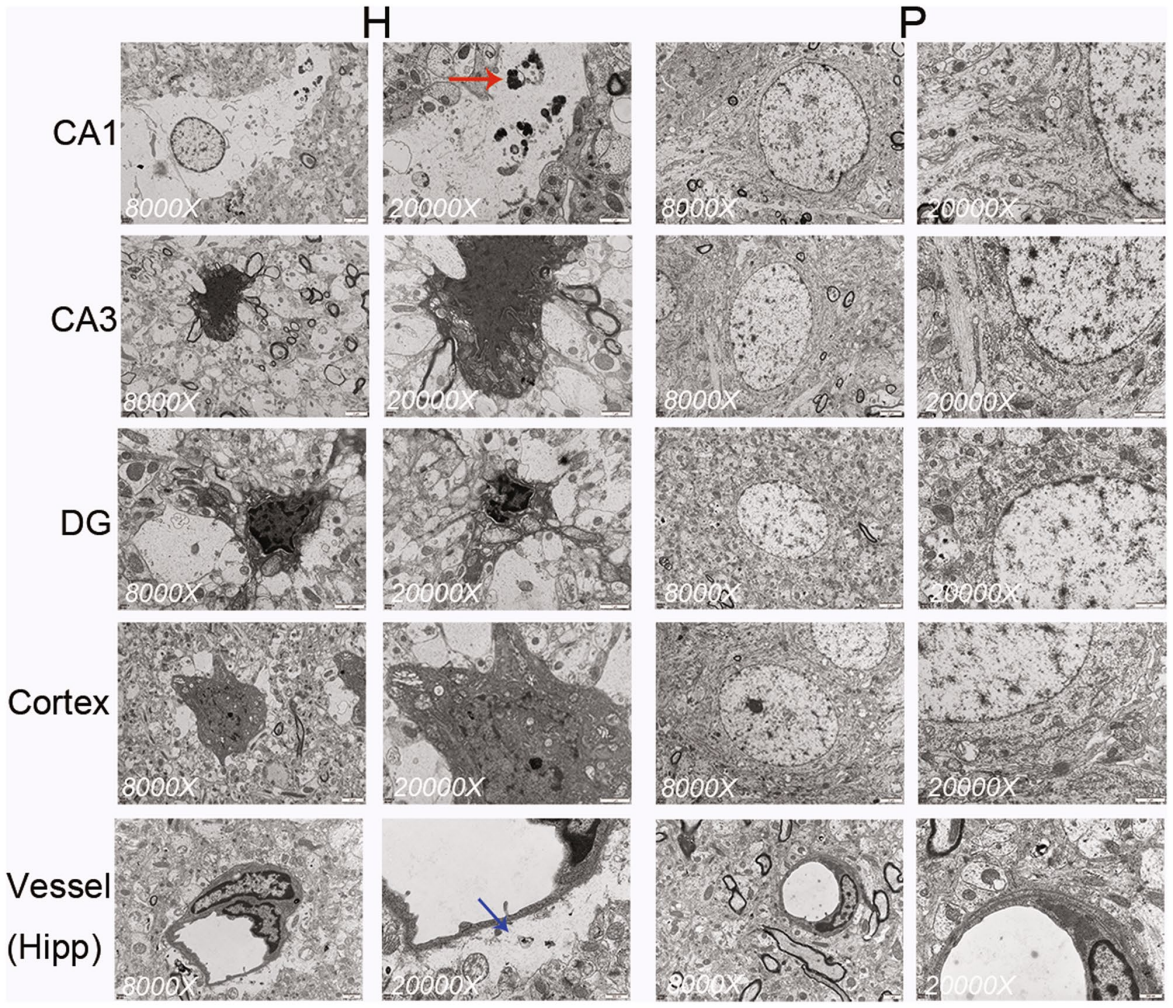
Plateau hypoxia-induced neuroapoptosis and autophagy (as indicated with red arrows) in the hippocampus and cortex of rats (n = 3 per group). Widespread perivascular spaces were seen in the hippocampus (blue arrows). Scale bar = 100 μm

H group rats showed neuronal apoptosis in four regions, viz., CA1, CA3, and DG, in the hippocampus and the cortex (Fig. 12). Specifically, apoptotic death was characterized by increased electron density, nuclear chromatin condensation, shrinkage of the nuclear membrane and perinuclear organelle vacuolization, which was found in the neurons of H group rats. Additionally, the neurons and axons in the H group rats were mitochondrially swollen. The peripheral space in the vessels of the hippocampus of H group rats was widened. In contrast, no obvious ultrastructural abnormalities with neuronal cells and vessels were found in the above regions of the P group rats.

## Discussion

In the present study, we used multimodal MR imaging to detect morphological, molecular and microvascular alterations in the hippocampus in a rat model of chronic exposure to a hypoxic plateau environment. The results of the study show reduced rGMV, lower CrCEST contrast and higher transport coefficient Ktrans in the hippocampus of H group rats compared with that of the control, P group rats. Further histopathological examinations revealed neural degeneration and apoptosis, proliferation of glial cells and decreased spine density in the hippocampus of H group rats. Moreover, correlation analysis showed that quantitative MRI parameters of the hippocampus were associated with the behavioral performance of H group rats. These findings suggest that chronic exposure to a hypoxic plateau environment might adversely affect the gray matter, energy metabolism and microvascular permeability of the hippocampus of rats and thus contribute to deficits in affective and cognitive functions of H group rats.

Prior VBM studies have revealed that high-altitude residents with chronic exposure to hypobaric hypoxia showed significant structural modifications in the brain, which include a decrease in the regional cortical gray matter accompanied by changes in the white matter, which may underlie a cerebral adaptation to hypoxic exposure [13, 42, 43]. However, there has been little direct investigation into morphological changes in the entire brains of H group rats after long-term exposure to plateau hypobaric hypoxia. VBM analysis in this study revealed gray matter atrophy in the hippocampus of rats exposed to plateau hypobaric hypoxia for 8 months. These findings are consistent with a previous report that the bilateral hippocampus was one of the first affected regions in rats after high-altitude exposure [6]. Therefore, VBM may provide qualitative indices of hypoxic plateau-induced brain damage to the regional volume or density reductions of the gray matter on the voxel scale. rGMV reduction was also found in the primary somatosensory cortex of H group rats compared with that of P group rats in this study.

It has been reported that many tissue properties, such as local cell count, spatial arrangement of cells and cell type composition, may affect the GMV [44]. Hypobaric hypoxia can induce neurodegeneration, which results in decreased dendritic arborization [45, 46]. Decreased dendritic arborization can cause GMV reduction [47]. Previous reports showed that there was remarkable neuron loss after hypoxic challenge in the hippocampus [48]. Reduced rGMV, as demonstrated by structural MR imaging, can be used as a surrogate biomarker for neuronal losses [49, 50]. Thus, hypobaric hypoxia-induced changes in rGMV may be primarily related to neurodegeneration and death of cells in the hippocampus. Our correlation analysis revealed that plateau hypoxia exposure-induced learning deficits in the MWM test were directly associated with reduced rGMV in the hippocampus. Previous reports have come to similar conclusions that reduced rGMV in the hippocampus is associated with cognitive decline in patients with Parkinson’s disease [51] and in animals with chronic epilepsy [52]. Thus, rGMV obtained with the VBM analysis method can be used as a reliable biomarker of cognitive impairment in rats and humans.

CrCEST can significantly enhance the sensitivity to low concentrations of Cr through exchangeable protonsis [53]. Cr reaches its peak at approximately 2 ppm in the water saturation spectrum (Z-spectrum), whereas the effect of CrCEST depends linearly on Cr concentration in the physiological pH range [35]. The guanidinium protons in Cr and the amide and guanidine protons in phosphocreatine (PCr) are exchangeable with water. PCr produces adenosine triphosphate, which is a common currency of bioenergy, by losing phosphorus. As this process is reversible, Cr can be phosphorylated to form PCr, which is often used as an energy reserve to quickly produce large amounts of ATP. Therefore, it is essential to detect Cr and PCr for the detection of many energy-related diseases. Previous CrCEST studies of the brain [54], muscle [55] and myocardium [56] have provided promising data and evidence supporting the feasibility of CrCEST to freely detect Cr at millimolar physiological concentrations. Recent studies have reported a significant linear correlation between the testicular CrCEST effect and testicular ischemia time [47] and spermatogenesis [48]. Although 1H magnetic resonance spectroscopy (MRS) and 31P MRS can also detect Cr and PCr, their low detection sensitivity, poor spatial resolution and longer acquisition duration make it difficult for MRI imaging to detect free Cr signals with satisfactory precision [57]. In addition, most MRI instruments today are still not equipped with phosphorus coils specific to 31P MRS.

Cr concentration, measured by CrCEST in our study, could differentiate H group rats and P group rats and was significantly and positively correlated with neurological deficits. As Cr can be neuroprotective [58], its loss may be viewed as a reflection of neuronal cell death [59, 60]. Previous studies revealed that altered brain bioenergetics were observed in subjects recruited in cities of moderately elevated altitude [61]. Loss and depletion of energy metabolites in the hippocampus after chronic plateau hypoxia exposure might possibly be useful biomarkers of cognitive impairment in animals and humans.

A recent study has suggested that CrCEST is highly sensitive to subtle pH variations in the brains of patients with early Alzheimer’s disease [27]. It has been reported that neuroinflammation can cause intracellular cerebral pH reduction [62, 63]. Previous studies have revealed that high altitude is related to changes in the intracellular pH and inorganic phosphate levels of the brain [64–66]. However, due to the lack of a method to test intracellular pH, we cannot rule out the likelihood that the decreased CrCEST contrast is probably related to a decreased intracellular pH rather than the loss of Cr concentration or a combination of both. Therefore, it has to be elucidated in further studies how or whether neuroinflammation-triggered reduction of intracellular pH is related to the decrease in pH-dependent CrCEST contrast after chronic exposure to plateau hypoxia.

BBB integrity is usually maintained by three main elements, including brain microvascular endothelial cells, astrocytic end-feet and pericytes [67]. Brain microvascular endothelial cells play an essential role in maintaining the normal functions of the BBB primarily through the presence of tight junction proteins. Although dyes such as Evans blue are commonly used as biomarkers of brain barrier integrity, Evans blue is not satisfactory enough for the study of BBB dysfunction because it is not usable under most circumstances [68].

As DCE–MRI can specifically evaluate BBB integrity by measuring BBB leakage to quantify the extent of contrast extravasation to the brain, it has thus paved the way for further studies of the microcirculation in preclinical and clinical settings [69]. In addition to measuring BBB disruption in tumors, multiple sclerosis and acute ischemic stroke, DCE–MRI has recently been used to measure more subtle and chronic BBB disruptions, as observed in neurodegenerative diseases such as Alzheimer’s disease and Parkinson’s disease [70, 71].

Previous studies have reported that chronic exposure to hypobaric hypoxia causes apparent anatomical variations in the BBB [72, 73] and diminishes the integrity of the BBB [74, 75]. Ktrans, an important metric of DCE–MRI and a constant of transfer, is widely used to evaluate BBB permeability in vivo. Ktrans is also a complicated combination of the tissue perfusion flow rate with permeability-surface area per volume of tissue. In the present study, we discovered that compared with P group rats, H group rats had dramatically higher Ktrans values estimated by the extended Tofts model in the hippocampus and the cortex, which is indicative of an increased BBB permeability in these animals. A perivascular lake formed by fluid extravasation was observed under an electronic microscope, which might be characteristic of vasogenic edema [73]. Therefore, based on a DCE–MRI study, the results of our study indicate that chronic exposure to plateau hypoxia may increase BBB permeability in the hippocampus and thus compromise the integrity of the BBB. Disruptions of the BBB in the hippocampus have been reported to probably contribute to cognitive impairment [76, 77]. This may plausibly provide further data that support the claim that cognitive impairment is caused by high-altitude hypoxia.

High-altitude exposure may induce chronic neuroinflammation and microglial activation [74, 78], which may damage the BBB. With chronic exposure of H group rats to hypobaric hypoxia, proinflammatory cytokines in their plasma and brain can be upregulated [79], which might be primarily responsible for BBB disruption [80]. In view of this, we suggest that quantitative parameters derived from DCE–MRI can be used as effective biomarkers of BBB damage [36, 81] associated with neuroinflammation [82].

After 8 months of exposure to plateau hypoxia, oxidative stress, chronic neuroinflammation, neuronal degeneration and apoptosis were observed in the hippocampal tissues of H group rats, which may be indicative of continued hypoxic brain damage. In addition, increased proinflammatory cytokine levels and white blood cell counts in the blood of H group rats were also observed, which may be indicative of systemic inflammation after long-term exposure to a high-altitude environment. Chronic neuroinflammation may cause alterations to energy metabolism and BBB permeability in the hippocampus and decrease hippocampal spine density. There has been considerable evidence that neuroinflammatory responses and oxidative damage can affect hippocampal neurons, segue into measurable gray matter loss and eventually lead to cognitive disorders over time [83]. Therefore, we suggest that long-term plateau hypoxia exposure may further wear out the brain structure and physical and pathophysiological functions of H group rats.

Several limitations in this study should be acknowledged. First, it utilized only single-slice CrCEST and double-slice DCE imaging in the gross hippocampus region, whereas multiple cross-sectional CrCEST and DCE imaging analyses are needed to further validate the conclusion of this study. Second, CEST acquisitions were performed under anesthesia with isoflurane, whose effect on Cr levels in the hippocampus of the rats is not to be neglected [84]. Third, we chose male rats as the experimental model to exclude the influencing factors of fluctuations in reproductive hormones, as estrogen appears to affect sensitivity to hypoxia-induced hippocampal damage in female rats [85]. In addition, female rats were not used in this study to reduce variability due to endocrine cycles. Thus, we used only male rats for behavioral consistency. Finally, we used a Sprague–Dawley male animal model of chronic plateau hypoxia that exhibited genetic variability in a plateau environment after prolonged hypoxia exposure, whereas other animals, such as canines and nonhuman primates or mice, were not utilized but should be used in future studies.

## Conclusions

Our study demonstrated hippocampal damage in a rat model after chronic exposure for 8 months to a hypoxic plateau environment. Multimodal noninvasive MR imaging (VBM, CrCEST, and DCE), by measuring alterations in rGMV, CrCEST contrast and Ktrans values, revealed hippocampal atrophy, decreased Cr concentration and increased BBB permeability in H group rats compared with control rats. Further correlation analysis shows that the altered MR imaging parameters in the hippocampus of H group rats were associated with cognitive dysfunctions. In summary, these findings suggest that multimodal MR imaging techniques can be used as effective means to detect plateau hypoxia-induced hippocampal damage and provide potential biomarkers for understanding the mechanism underlying plateau hypoxia-mediated affective and cognitive dysfunctions.

## Supplementary Information

Below is the link to the electronic supplementary material.

Fig. S1 T2-weighted images of one rat from the H group before data processing (a, e). Tissue probabilistic maps for gray matter (b, f), white matter (c, g), and cerebrospinal fluid (CSF) (d, h) images after voxel-based morphometry segmentation. Supplementary file1 (TIF 3179 KB)

Fig. S2 ROI definition in the right hippocampus and cortex of rats by dynamic contrast-enhanced MR imaging. a. The reference positions of slice 1 and slice 2 in the sagittal plane. Slice 1 was defined at bregma -2.36 mm, and slice 2 was defined at bregma -4.70 mm (a). The coordinates of the slices are represented in relation to the anterior commissure. The hippocampus (ROI 1) and cortex (ROI 2) in slice 1 are shown on coronal T2WI (b). The hippocampus (ROI 3) and cortex (ROI 4) in slice 2 are shown on coronal T2WI (c). ROI = region of interest, T2WI = T2-weighted imaging, R = right. Supplementary file2 (TIF 1464 KB)

Supplementary file3 (DOCX 32 KB)
