# Supplementary figures and images for: A Multimodal MR Imaging Study of the Effect of Hippocampal Damage on Affective and Cognitive Functions in a Rat Model of Chronic Exposure to a Plateau Environment
Source: Neurochem Res. 2022 Jan 4;47(4):979–1000. doi: 10.1007/s11064-021-03498-5 (PMC8891211; doi:10.1007/s11064-021-03498-5)

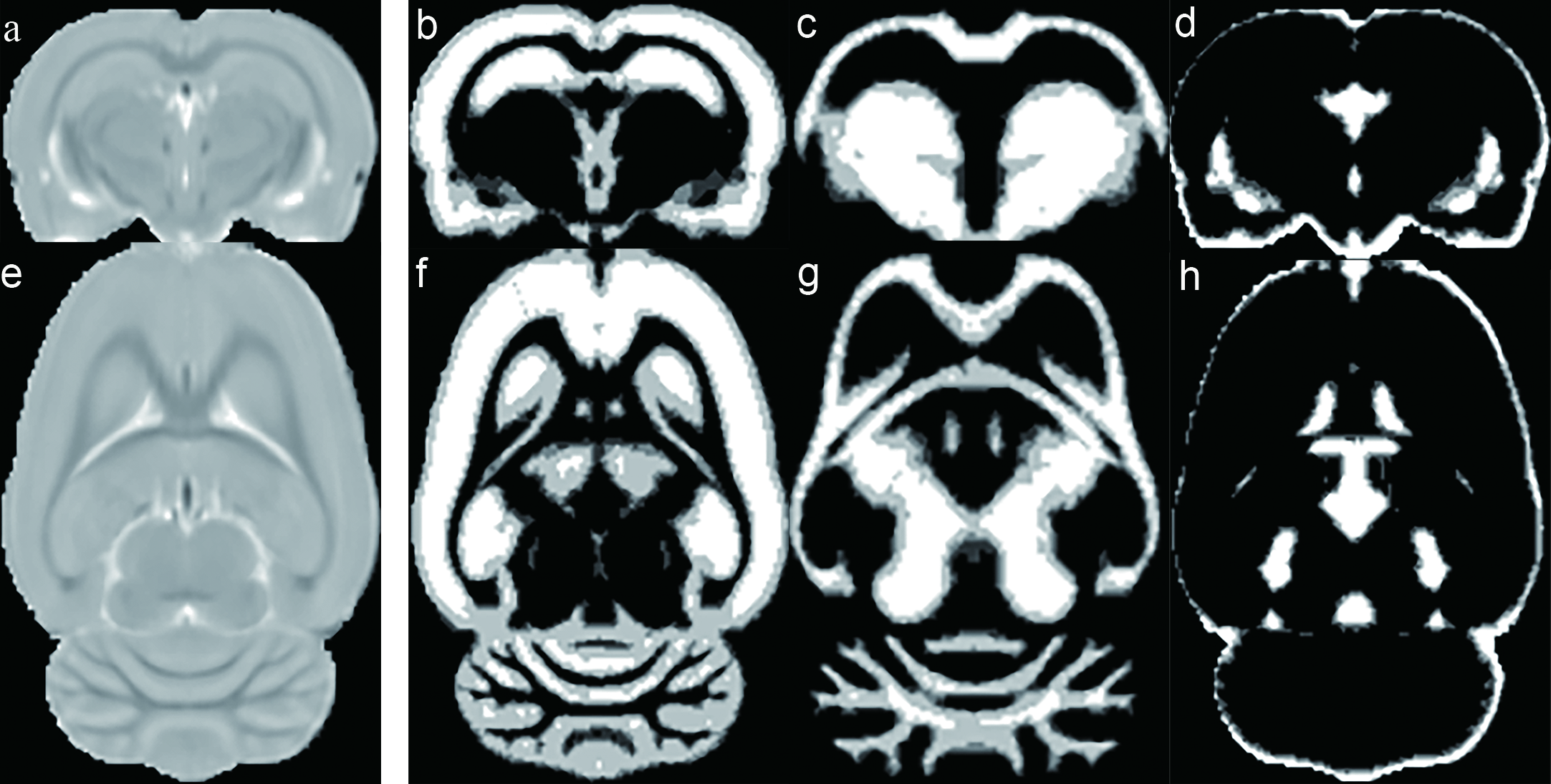

Supplement: Supplementary file 1 — Fig. S1 T2-weighted images of one rat from the H group before data processing (a, e). Tissue probabilistic maps for gray matter (b, f), white matter (c, g), and cerebrospinal fluid (CSF) (d, h) images after voxel-based morphometry segmentation. Supplementary file1 (TIF 3179 KB) [file 11064_2021_3498_MOESM1_ESM.tif]

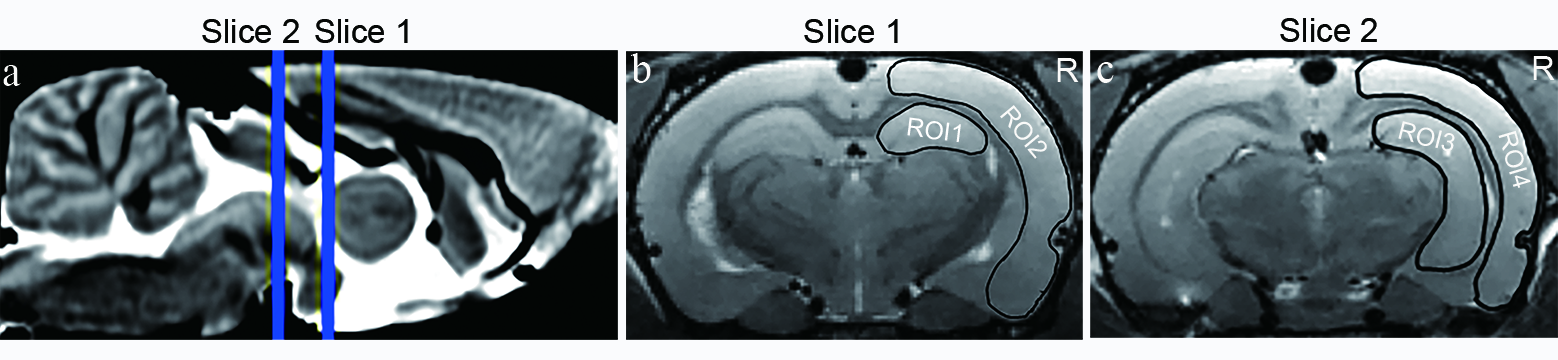

Supplement: Supplementary file 2 — Fig. S2 ROI definition in the right hippocampus and cortex of rats by dynamic contrast-enhanced MR imaging. a. The reference positions of slice 1 and slice 2 in the sagittal plane. Slice 1 was defined at bregma -2.36 mm, and slice 2 was defined at bregma -4.70 mm (a). The coordinates of the slices are represented in relation to the anterior commissure. The hippocampus (ROI 1) and cortex (ROI 2) in slice 1 are shown on coronal T2WI (b). The hippocampus (ROI 3) and cortex (ROI 4) in slice 2 are shown on coronal T2WI (c). ROI = region of interest, T2WI = T2-weighted imaging, R = right. Supplementary file2 (TIF 1464 KB) [file 11064_2021_3498_MOESM2_ESM.tif]
