# Supplementary material for: A Multimodal MR Imaging Study of the Effect of Hippocampal Damage on Affective and Cognitive Functions in a Rat Model of Chronic Exposure to a Plateau Environment
Source: Neurochem Res. 2022 Jan 4;47(4):979–1000. doi: 10.1007/s11064-021-03498-5 (PMC8891211; doi:10.1007/s11064-021-03498-5)
Supplement: Supplementary file 3 — Supplementary file3 (DOCX 32 KB) [file 11064_2021_3498_MOESM3_ESM.docx]

Supplementary Table S1 Further details of statistical analysis in this study

| Figure  number | n | Normality  test | Equal  variance  test | Statistic  method | P value | *F/t/r* value | Post hoc  multiple  comparisons  test |
| --- | --- | --- | --- | --- | --- | --- | --- |
| 2a | n = 6 per group | failed | failed | Mann-Whitney  Rank Sum  Test (two-tailed) | 0.0022 | U = 0 |  |
| 2b | n = 6 per group | passed | passed | Unpaired t  test (two-tailed) | 0.0258 | t_10_ = 2.615 |  |
| 2c | n = 6 per group | passed | passed | Unpaired t  test (two-tailed) | 0.0108 | t_10_ = 3.122 |  |
| 2d | n = 6 per group | failed | passed | Mann-Whitney  Rank Sum  Test (two-tailed) | 0.0411 | U = 5 |  |
| 2e | n = 6 per group | passed | passed | Unpaired t  test (two-tailed) | 0.0406 | t_10_ = 2.350 |  |
| 2f | n = 6 per group | passed | passed | Unpaired t  test (two-tailed) | 0.0044 | t_10_ = 3.662 |  |
| 2g | n = 6 per group | passed | passed | Unpaired t  test (two-tailed) | 0.0381 | t_10_ = 2.388 |  |
| 2h | n = 6 per group | passed | failed | Mann-Whitney  Rank Sum  Test (two-tailed) | 0.0022 | U = 0 |  |
| 2i | n = 6 per group | failed | failed | Mann-Whitney  Rank Sum  Test (two-tailed) | 0.0022 | U = 0 |  |
| 3a | n = 12 per group | failed | passed | Mann-Whitney  Rank Sum  Test (two-tailed) | 0.0261 | U = 34 |  |
| 3b | n = 12 per group | passed | passed | Unpaired t  test (two-tailed) | 0.4507 | t_22_ = 0.7680 |  |
| 3c | n = 12 per group | failed | passed | Mann-Whitney  Rank Sum  Test (two-tailed) | 0.0068 | U = 26 |  |
| 3d | n = 12 per group | failed | failed | Mann-Whitney  Rank Sum  Test (two-tailed) | 0.0085 | U = 28 |  |
| 3e | n = 12 per group | passed | passed | Unpaired t  test (two-tailed) | 0.0587 | t_22_ = 1.994 |  |
| 3f | n = 12 per group | passed | passed | Unpaired t  test (two-tailed) | 0.6049 | t_22_ = 0.5248 |  |
| 3g | n = 12 per group | failed | failed | Mann-Whitney  Rank Sum  Test (two-tailed) | 0.0847 | U = 42 |  |
| 3h | n = 12 per group | passed | passed | Two-way  RM ANOVA  with Sidak’s  post hoc  comparison | 0.044 (Interaction)  < 0.0001 (day)  < 0.0001 (group) | group (F(1, 22) = 29.35)  day (F(3.142, 69.12) = 28.32)  group* day (F(4, 88) =2.56) | *P* = 0.7363 (Day1)  *P* = 0.5645 (Day2)  *P* = 0.2935 (Day3)  *P* < 0.0001 (Day4)  *P* < 0.0001 (Day5) |
| 3i | n = 12 per group | passed | passed | Two-way  RM ANOVA  with Sidak’s  post hoc  comparison | 0.039 (Interaction)  < 0.0001 (day)  < 0.0001 (group) | group (F(1, 110) = 27.10)  day (F(3.075, 67.64) = 31.54)  group* day (F(4, 88)= 2.63) | *P* = 0.9995 (Day1)  *P* = 0.3326 (Day2)  *P* = 0.2691 (Day3)  *P* = 0.0207 (Day4)  *P* < 0.0001 (Day5) |
| 3j | n = 12 per group | failed | passed | Mann-Whitney  Rank Sum  Test (two-tailed) | 0.0024 | U = 21.50 |  |
| 3k | n = 12 per group | failed | passed | Mann-Whitney  Rank Sum  Test (two-tailed) | 0.0479 | U = 38.50 |  |
| 5d | n = 12 per group | passed | passed | Unpaired t  test (two-tailed) | <0.0001 | t_22_ = 6.906 |  |
| 5e | n = 12 per group | failed | passed | Mann-Whitney  Rank Sum  Test (two-tailed) | 0.0233 | U = 26 |  |
| 6c | n = 12 per group | passed | passed | Unpaired t  test (two-tailed) | <0.0001 | t_22_ = 6.546 |  |
| 6d | n = 12 per group | passed | failed | Mann-Whitney  Rank Sum  Test (two-tailed) | 0.0043 | U = 24 |  |
| 7a | n = 12 | passed |  | Pearson’s correlation coefficient | 0.0239 | *r* = 0.6437 |  |
| 7b | n = 12 | passed |  | Pearson’s correlation coefficient | 0.0327 | *r* = 0.5707 |  |
| 7c | n = 12 | passed |  | Pearson’s correlation coefficient | 0.0378 | *r* = 0.6034 |  |
| 7d | n = 12 | passed |  | Pearson’s correlation coefficient | 0.0136 | *r* = 0.6868 |  |
| 7e | n = 12 | passed |  | Pearson’s correlation coefficient | 0.0347 | *r* = 0.6113 |  |
| 7f | n = 12 | passed |  | Pearson’s correlation coefficient | 0.0251 | *r =* 0.6521 |  |
| 7g | n = 12 | passed |  | Pearson’s correlation coefficient | 0.0014 | *r =* *0.8097* |  |
| 7h | n = 12 | passed |  | Pearson’s correlation coefficient | 0.0068 | *r =* 0.7324 |  |
| 7i | n = 12 | passed |  | Pearson’s correlation coefficient | 0.0077 | *r = -0.7243* |  |
| 7j | n = 12 | passed |  | Pearson’s correlation coefficient | 0.0113 | *r =* -0.7000 |  |
| 7k | n = 12 | passed |  | Pearson’s correlation coefficient | 0.0268 | *r =* -0.6341 |  |
| 7l | n = 12 | passed |  | Pearson’s correlation coefficient | 0.0180 | *r =* -0.6660 |  |
| 8d | n = 6 per group | passed | failed | Mann-Whitney  Rank Sum  Test (two-tailed) | <0.0001 | U = 3.5 |  |
|  |  | passed | failed | Mann-Whitney  Rank Sum  Test (two-tailed) | <0.0001 | U = 2.5 |  |
|  |  | passed | failed | Mann-Whitney  Rank Sum  Test (two-tailed) | <0.0001 | U = 1.5 |  |
| 9b | n = 6 per group | passed | passed | Unpaired t  test (two-tailed) | 0.0068 | t_10_ = 3.619 |  |
|  |  | passed | passed | Unpaired t  test (two-tailed) | <0.0001 | t_10_ = 9.544 |  |
|  |  | passed | passed | Unpaired t  test (two-tailed) | 0.0203 | t_10_ = 2.887 |  |
| 9d | n = 6 per group | passed | passed | Unpaired t  test (two-tailed) | 0.0032 | t_10_ = 3.844 |  |
|  |  | passed | failed | Mann-Whitney  Rank Sum  Test (two-tailed) | 0.0476 | U = 5.5 |  |
|  |  | passed | passed | Unpaired t  test (two-tailed) | 0.0373 | t_10_ = 2.401 |  |
| 10b | n = 6 per group | passed | passed | Unpaired t  test (two-tailed) | 0.0043 | t_22_ = 3.179 |  |
|  |  | passed | passed | Unpaired t  test (two-tailed) | 0.0017 | t_10_ = 3.563 |  |
|  |  | passed | passed | Unpaired t  test (two-tailed) | 0.0316 | t_10_ = 2.296 |  |
|  |  | passed | passed | Unpaired t  test (two-tailed) | 0.0131 | t_10_ = 2.656 |  |
| 10d | n = 6 per group | passed | passed | Two-way  RM ANOVA  with Sidak’s  post hoc  comparison | < 0.0001 (Interaction)  < 0.0001 (distance)  = 0.0085 (group) | group (F(1, 94)= 7.218)  distance (F(2.429, 228.4)= 73.98)  group* distance (F(9, 846) = 4.151) | *P* = 0.0083 (10μm)  *P* =0.0002 (20μm)  *P* =0.0147 (30μm)  *P* =0.0172 (40μm)  *P* =0.0036 (50μm)  *P* =0.4997 (60μm)  *P* =0.1433 (70μm)  *P* =0.5169 (80μm)  *P* > 0.9999 (90μm)  *P* = 0.9691 (100μm) |
|  |  | passed | passed | Two-way  RM ANOVA  with Sidak’s  post hoc  comparison | < 0.0001 (Interaction)  < 0.0001 (distance)  = 0.0193 (group) | group (F(1, 94)= 5.671)  distance (F(1.265, 118.9)= 88.18)  group* distance (F(9, 846) = 4.148) | *P* = 0.0167 (10μm)  *P* =0.0011 (20μm)  *P* =0.0399 (30μm)  *P* =0.0327 (40μm)  *P* =0.0269 (50μm)  *P* =0.1433 (60μm)  *P* =0.5250 (70μm)  *P* =0.9982 (80μm)  *P* = 0.9984 (90μm)  *P* = 0.9943 (100μm) |
|  |  | passed | passed | Two-way  RM ANOVA  with Sidak’s  post hoc  comparison | = 0.0288 (Interaction)  < 0.0001 (distance)  = 0.0229 (group) | group (F(1, 94)= 5.350)  distance (F(2.169, 203.9)= 27.85)  group* distance (F(9, 846) = 1.205) | *P* = 0.7695 (10μm)  *P* =0.9998 (20μm)  *P* =0.6583 (30μm)  *P* =0.9430 (40μm)  *P* =0.8431 (50μm)  *P* =0.6885(60μm)  *P* =0.2898 (70μm)  *P* =0.6769 (80μm)  *P* = 0.4351 (90μm)  *P* = 0.0013 (100μm) |
|  |  | passed | passed | Two-way  RM ANOVA  with Sidak’s  post hoc  comparison | = 0.0299 (Interaction)  < 0.0001 (distance)  = 0.0022 (group) | group (F(1, 94)= 9.931)  distance (F(1.716, 161.3)= 70.54)  group* distance (F(9, 846) = 2.068) | *P* = 0.0004 (10μm)  *P* =0.0029 (20μm)  *P* =0.0062 (30μm)  *P* =0.0001 (40μm)  *P* =0.6794 (50μm)  *P* =0.6952 (60μm)  *P* =0.8790 (70μm)  *P* =0.5002 (80μm)  *P* = 0.7885 (90μm)  *P* > 0.9999 (100μm) |
| 11b | n = 6 per group | failed | passed | Mann-Whitney  Rank Sum  Test (two-tailed) | 0.0022 | U = 0 |  |
|  |  | failed | failed | Mann-Whitney  Rank Sum  Test (two-tailed) | 0.0022 | U = 0 |  |
|  |  | passed | passed | Unpaired t  test (two-tailed) | 0.0005 | t_10_ = 5.614 |  |
